# Immunoglobulin constant regions provide stabilization to the paratope and enforce epitope specificity

**DOI:** 10.1016/j.jbc.2024.107397

**Published:** 2024-05-18

**Authors:** Scott A. McConnell, Arturo Casadevall

**Affiliations:** Department of Molecular Microbiology and Immunology, Johns Hopkins Bloomberg School of Public Health, Baltimore, Maryland, USA

**Keywords:** immunoglobulins, single-chain variable fragments, epitope mapping, molecular dynamics, *Cryptococcus neoformans*, fungal pathogens

## Abstract

Constant domains in antibody molecules at the level of the Fab (C_H_1 and C_L_) have long been considered to be simple scaffolding elements that physically separate the paratope-defining variable (V) region from the effector function-mediating constant (C) regions. However, due to recent findings that C domains of different isotypes can modulate the fine specificity encoded in the V region, elucidating the role of C domains in shaping the paratope and influencing specificity is a critical area of interest. To dissect the relative contributions of each C domain to this phenomenon, we generated antibody fragments with different C regions omitted, using a set of antibodies targeting capsular polysaccharides from the fungal pathogen, *Cryptococcus neoformans*. Antigen specificity mapping and functional activity measurements revealed that V region-only antibody fragments exhibited poly-specificity to antigenic variants and extended to recognition of self-antigens, while measurable hydrolytic activity of the capsule was greatly attenuated. To better understand the mechanistic origins of the remarkable loss of specificity that accompanies the removal of C domains from identical paratopes, we performed molecular dynamics simulations which revealed increased paratope plasticity in the scFv relative to the corresponding Fab. Together, our results provide insight into how the remarkable specificity of immunoglobulins is governed and maintained at the level of the Fab through the enforcement of structural restrictions on the paratope by C_H_1 domains.

Bivalent antibodies (Abs) are composed of two ‘Fragment antigen binding’ arms (Fab) joined by one ‘Fragment crystallizable’ (Fc) stem. The Fc is a homodimer composed exclusively of constant domains, while the Fab is a heterodimer of constant (C) and variable (V) domains. V domains collaborate to form dimeric binding surfaces, called paratopes, that determine the specificity of the antibody. Paratope specificity is driven primarily by complementarity-determining region (CDR) loops, which are supported by framework regions in the V domains. In mice and humans, V domains are encoded by a vast gene repertoire and are much more divergent in sequence than C domains on the germline level. During the genetic assembly of antibodies, somatic recombination and subsequent targeted hypermutation to CDRs generate additional structural and functional diversity (1). In contrast, the primary sequences of C domains within isotype families are highly conserved and are located on distinct genetic loci from the V gene elements.

The supramolecular arrangement of the resultant immunoglobulin consists of two heavy and two light chains crosslinked into a highly stabilized Y-shaped structure. Enzymatic digestion with papain separates the Ab into Fab and Fc fragments (2). The Fab arms consist of the light and heavy chain V domains (V_H_ and V_L_) and C_H_1 and C_L_ constant domains which are covalently associated by a C-terminal disulfide linkage. Limited structural flexibility exists between the V and C domains in the Fab, known as the “elbow angle” (3, 4, 5). The Fc homodimer is formed by the remaining C domains of the heavy chain (C_H_2 and C_H_3, for the IgG isotype) (6). A flexible “hinge” region with additional disulfide crosslinks lies at the junction between Fab and Fc, allowing for independent motion of each Fab arm. The C domains mediate important functions such as complement activation, Fc receptor engagement, and half-life extension (7). From a functional standpoint, Ab neutralization can be Fab-driven, *via* direct blocking of receptor or cellular process; or Fc-driven, in which case the Ab tags an antigenic target for destruction through effector functions. Therefore, the common consensus of Abs as modular molecules comprised of two primary regions of independent functionality has been the working paradigm in immunology since classic studies of antibody proteolysis by Porter in the late 1950s (2). However, in the past decade, the theory of independent functionality of V and C domains has come into question. While Fabs generally retain comparable affinity to the parental mAb (8), corresponding single-chain Fvs have been reported to vary in affinity (9, 10), suggesting that C domains in the Fab play some role in Ag binding. Further, multiple groups have reported differences in affinity and/or specificity of engineered antibodies with identical V domains but different isotypes (11, 12, 13, 14, 15). The most parsimonious explanation for the observed modulation in affinity and selectivity is that C domains of different isotypes have subtle effects on the structure and thus binding parameters of the V domains (16).

Given the growing evidence that C domains can play a significant role in Ag binding, we sought to obtain greater insight into this phenomenon by investigating the influence of C domains on the specificity of antibody fragments derived from antibodies directed to the glucuronoxylomannan (GXM) polysaccharide Ag in the capsule of *Cryptococcus neoformans*. The capsule of *C. neoformans*, an important virulence factor, protects the microbe from desiccation, phagocytosis, and modulates immune response (17, 18). The capsule is a complex structure comprised of GXM polymers with a distribution of motifs that varies between strains and serotypes (19, 20, 21). mAbs generated against capsular material often manifest distinct specificity to different GXM motifs which determines their protective efficacy (22, 23, 24). Through studying antibody fragments of three well-characterized protective mAbs against *C*. *neoformans* capsular polysaccharide (mAbs 18B7, 2H1, and 3E5), we gleaned important insights into the relative contributions of C domains on the affinity and specificity of the resultant constructs. Here, we demonstrate that the C_H_1 domain plays an important role in determining the specificity of the three mAbs surveyed for GXM Ag through a combinatorial analysis based on antibody fragment localization on the cryptococcal capsules, epitope mapping using a panel of native exopolysaccharide and synthetic glycans of different motifs, and molecular dynamics simulations of Fv plasticity.

## Results

### Generation and validation of scFv fragments

**Figure 1:**
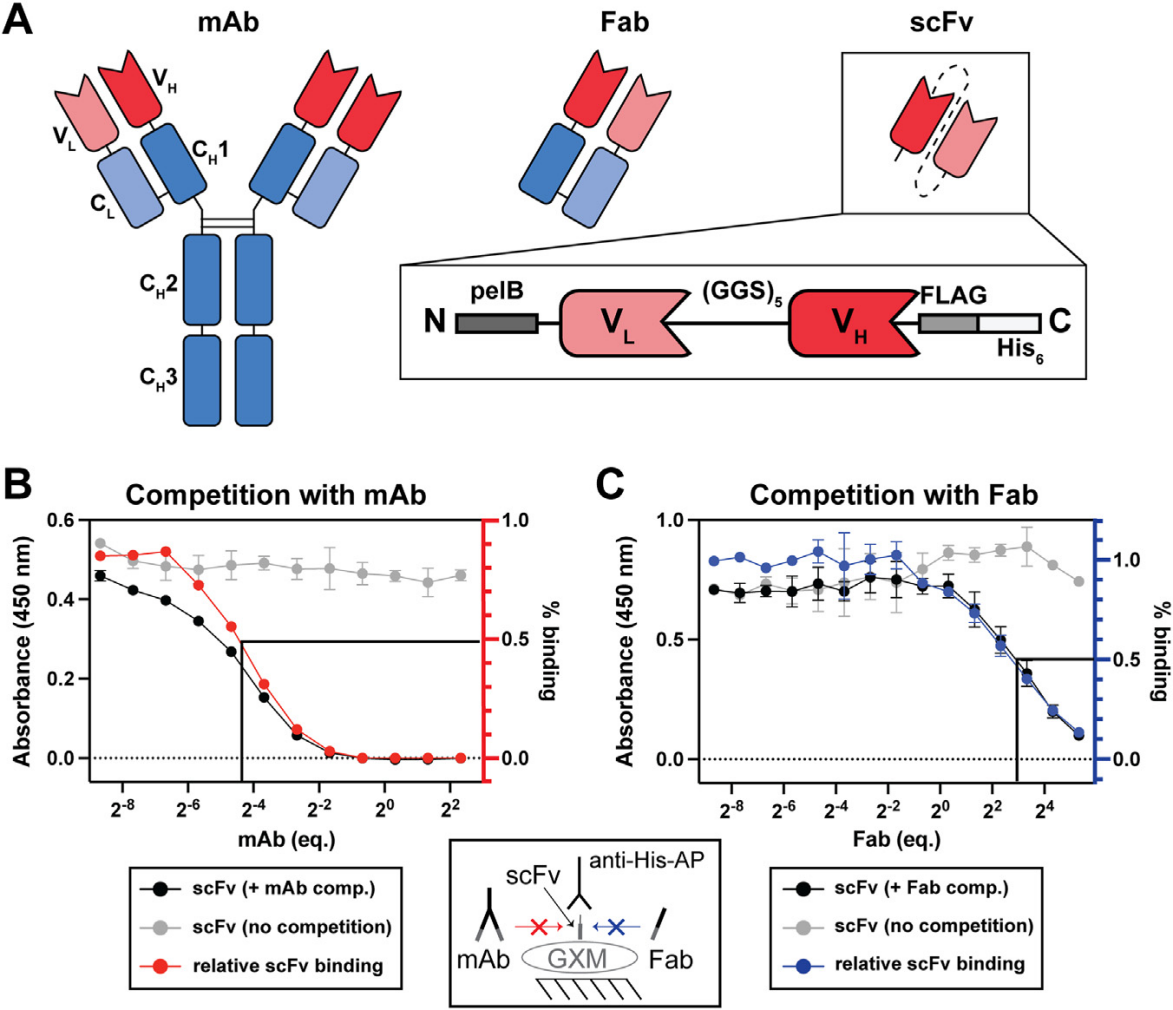
**Single-chain Fv antibody fragment 18B7 competes for binding to GXM antigen with corresponding mAb.***A*, schematic depicting the antibody fragments used in this study. Variable domains are colored *red* and constant domains in *blue*. Light chains are a lighter hue than heavy chains. *Inset*, map of scFv constructs used in this study: domains are in the V_L_-V_H_ arrangement, connected by a 15-residue glycine-serine linker with FLAG and His_6_ peptide tags are appended to the C-terminus. *B* and *C*, competition for binding of mAb or Fab 18B7 and scFv 18B7 to *C. neoformans* strain H99 exopolysaccharide was assessed by ELISA competition experiments. In both experiments, a constant concentration of scFv competed for binding with an increasing concentration gradient of mAb or Fab 18B7 and bound scFv was measured at each point to determine binding inhibition. Data points for scFv binding with and without competition are colored *black*, *gray*. Data represent the mean ± SD of two replicate measurements. The molar equivalents of mAb or Fab that achieved 50% binding inhibition are indicated on the plots. *B*, the binding of antigen by scFv 18B7 at a constant concentration with respect to a gradient of competing mAb 18B7 was determined. The percentage of uninhibited scFv binding to antigen at each mAb concentration is plotted in *red*. *C*, the binding of antigen by scFv 18B7 at a constant concentration with respect to a gradient of competing Fab 18B7 was determined. The percentage of uninhibited scFv binding to antigen at each Fab concentration is plotted in *blue*.

To generate antibody fragments for this study, the primary sequences of mAbs 18B7, 2H1, and 3E5 (24, 25) were used to construct the single-chain V fragments (scFv) for each corresponding mAb. The V domains of these mAbs differ only at a few amino acid positions (Fig. S1), but they exhibit differences in fine specificity (26, 27, 28, 29). scFv constructs were engineered in a V_H_-linker-V_L_ format with a 15-residue (Gly_4_Ser)_3_ spacer between V_L_ and V_H_ domains to allow appropriate intramolecular domain-pairing and favor monomeric scFv formation (30, 31, 32). The C-terminus was engineered with tandem FLAG and hexahistadine tags to enable affinity purification immunoassay detection. A *pelB* leader sequence was appended to the N-terminus to target the protein to the periplasmic space for expression, where endogenous bacterial redox enzymes can facilitate proper disulfide bond formation (33, 34, 35) (Fig. 1). Soluble scFv was produced in high quantities in the periplasm, but primarily in the domain-swapped dimer form (Fig. S2*A*). Attempts to separate monomeric scFv from domain-swapped dimer were unsuccessful, so we instead purified the scFv from inclusion bodies. Refolding and re-oxidization under dilute conditions promoted proper intramolecular disulfide bond formation and yielded homogenous, monomeric refolded scFv preparations (Fig. S2*B*). Circular dichroism revealed that refolded scFv 18B7 retained the expected beta-character of the native immunoglobulin fold, based on the characteristic negative band at 218 nm and positive band at 195 nm. Unfolded proteins exhibit negative ellipticity at 195 nm and no significant ellipticity above 210 nm (36), and neither of those spectral signatures was observed. The unique signal at 230 nm is commonly found in β-proteins with closely packed aromatic sidechains or disulfide linkages (36, 37, 38) and is shared amongst the 18B7 family Ab fragments, suggesting that the native fold is maintained in scFv (Fig. S2*C*). This signal is more pronounced in the scFv and Fab, probably reflecting the increased contribution to the overall CD spectrum of interacting aromatic sidechains localized to the paratope (36, 37, 38). Furthermore, the elution times from size-exclusion chromatography of the 18B7 mAb, Fab, and scFv families follow the expected pattern for molecules of decreasing hydrodynamic radius. The absolute molecular weight of scFv 18B7 derived from multi-angle light scattering also suggests a monomeric species, with no evidence of higher-order oligomerization (Fig. S2*D*). These results together with evidence of antigen specificity of the scFv (see below) support the notion that the recombinant paratopes assumed their correct configuration.

After expression and purification of the scFv fragments, functional binding of GXM Ag was established for scFv 18B7 by competition ELISA. For mAb 18B7, the parental antibody and scFv competed for the same Ag, but 50% inhibition was only achieved at a ratio of nearly 16 equivalents of scFv to 1 eq of mAb (Fig. 1*B*). Given that the mAb is a bivalent binder, and avidity often drives antibody binding to polymeric glycan Ags (39), we also assessed the competition between monovalent scFv and Fab 18B7. Under the same conditions, 50% inhibition of scFv binding was observed at a molar ratio of 0.125 eq of scFv to 1 eq of Fab. Overall, the competition observed between scFv 18B7 and its corresponding mAb and Fab indicates that specificity for GXM was maintained. The affinity enhancement derived from the dual engagement of Fvs to the polymeric Ag in the case of the bivalent mAb rendered it a much more potent competitor than Fab.

### Staining patterns to single-motif capsules reveal differences in specificity and penetrance

After confirming that GXM-specific recombinant scFv fragments retained affinity for the same exopolysaccharide Ag as their parental mAbs, we investigated the binding of capsular polysaccharides on cryptococcal cells expressing a single GXM motif. The major component of capsular and secreted polysaccharide from *C. neoformans* is a repeating glycan polymer composed of a mannose backbone decorated with β-1,2 and β-1,4 linked xylose and mannose substituents (17, 40). Six different GXM motifs (trimannose polymer sections with common substituent arrangements) were identified by previous NMR analysis of the structural reporter groups of exopolysaccharide harvested from a range of cryptococcal strains (20). *C. neoformans* strains can express one or more GXM motifs. For this study, we selected strains 24067, Mu-1, 409B, and KT24066 as single motif strains comprised of exclusively GXM Motifs 1, 2, 3, and 4 (M1-M4), respectively (20), to assess binding to antigenically homogenous capsules.

**Figure 2:**
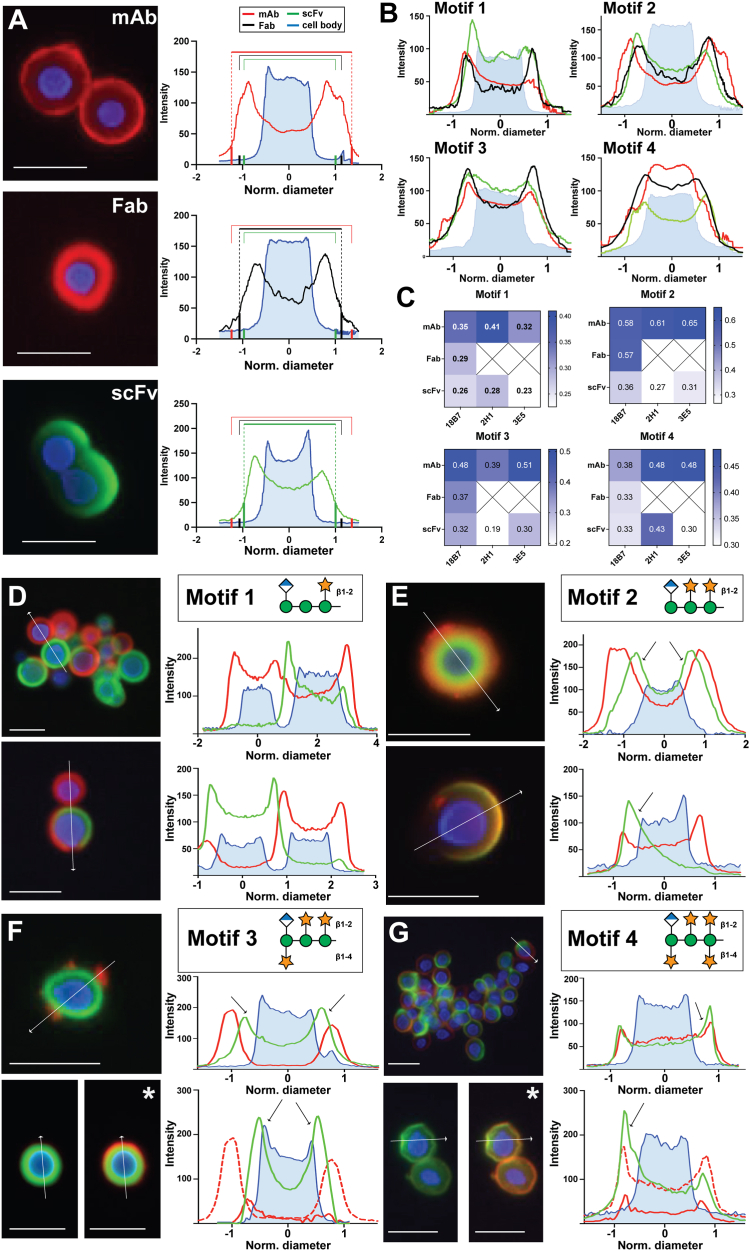
**Quantification of mono- and dual-staining of single motif capsules.** Capsules of each single motif strain were stained individually by each antibody fragment from the three Ab families (18B7, 2H1 and 3E5) and antibody staining patterns were analyzed by immunofluorescence. *A*, *left*: representative immunofluorescence images for the 18B7 family binding to strain Mu-1 (Motif 2). *Right*: pixel intensities of 1D slices of fluorescence for the representative cells. The diameter of each cell was normalized in this analysis to compare the relative capsular penetrance of each antibody fragment. *B*, quantification of capsular binding distances with respect to the cell body for each antibody fragment are overlaid to demonstrate the differences in penetrance between mAb, Fab and scFv. Fluorescence intensity plots for mAbs, Fabs, scFv and cell body are colored *red*, *black*, *green*, and *blue*, respectively. The intensity plots represent the average of five measurements for each condition. *C*, summary of capsular penetrance measurements for all cryptococcal strains and antibody formats. Reliable measurables of capsular penetrance for weak binding Fabs could not be obtained. See Fig. S2 for representative immunofluorescence staining of each antibody fragment with each single motif strain. *D–G*, each single motif strain was co-stained with mAb 18B7 and scFv 18B7. mAb and scFv were incubated with cells at equal molarities of Fv. mAb and scFv localization on each capsule were visualized by TRITC (*red*) and FITC (*green*) fluorescence, respectively. Pixel intensities through 1D slices for each fluorescent channel are quantified and displayed to right of each image. *White arrows* represent the 1D slice that was quantified. *Black arrows* indicate clear examples of increased penetrance of scFv with respect to mAb. For each motif, the structure of the triad is presented next to the motif. The mannan backbone and xylose and glucuronic acid sidechains are represented as *green circles*, *orange stars*, and *blue-white diamonds*, respectively, according to Symbol Nomenclature for Glycans (SNFG). Representative immunofluorescence capsular staining patterns are displayed for single motif cryptococcal cells expressing (*D*) Motif 1, (*E*) Motif 2, (*F*) Motif 3, and (*G*) Motif 4. For images marked with *asterisks*, the exposure for the *red* channel was increased to visualize the localization of the relatively weak binding mAb. The intensity plots for the increased exposure images are shown as *dotted lines* and overlaid with the intensity plots taking at the same exposure for both mAb and scFv, shown with solid lines. See Fig. S3 for additional representative images of co-staining with the other mAb or Fab and scFv pairs. *White scale bars* on all microscopy images indicate 10 μm.

Each individual mAb and scFv generally bound with homogenous annular binding patterns to the capsules of the single motif strains. The M4 capsule was recognized well by mAb 18B7, but binding by mAbs 2H1 and 3E5 was very weak and often punctate. ScFv bound uniformly to every capsule type. Conversely, Fabs generally bound in diffuse or punctate patterns to capsules of the same strains. Fab localization was often too weak to detect at the exposure time used to visualize mAb and scFv binding but could be observed with longer exposure (Fig. S3). Beyond qualitative binding patterns, capsular penetrance differed markedly between antibody formats. When the fluorescence intensity across 1D slices of the images was analyzed, a clear correlation of capsular penetrance with a molecular size of the antibody format emerged, with smaller scFv fragments binding regions more proximal to the cell body than large mAbs. mAbs mainly bound at the distal periphery of a capsule, leaving a vacated zone between the binding fringe and the cell body. The outer ring of capsule binding for scFvs began closer to the cell body than that of mAbs and extends nearly to the cell body boundary. The binding pattern of Fabs displayed an intermediate pattern, with a smaller zone of exclusion, but more proximal binding edge than the mAb (Fig. 2, *A–C*). The apparent stratification of each fragment was greatest within the M2 and M3 capsules, likely due to the relatively large capsular diameters of those strains (Fig. 2*C*).

To directly observe the competitive binding between mAbs and corresponding scFv, we co-stained the same capsules and visualized the staining patterns. For the mAb-scFv 18B7 pair, M2 motif-dominant capsules were bound with roughly equivalent density, although the scFv again penetrated deeper into the inner layers of the capsule than mAb (Fig. 2*E*). *C. neoformans* strains expressing M1 and M4 motif-dominant capsules displayed unique exclusionary binding patterns. M1 capsules were stained either by mAb or by scFv, but not both (Fig. 2*D*). Individual M4 motif-dominant capsules were stained primarily on each face of the capsule by mAb or scFv, with very little overlap (Fig. 2*G*). We note that significant cell-to-cell capsular heterogeneity that was not apparent with visualization by mAb binding alone was revealed by co-staining with the new scFv reagents developed in this study. The origins of antigenic differences in the capsule of *C. neoformans* cells of the same strain will be investigated in a subsequent report. scFv 18B7 bound much more readily to the M3 capsule, almost entirely outcompeting mAb for binding. Sharp puncta were often observed for mAb 18B7 binding, distinct from the dense and proximal binding of scFv 18B7 (Fig. 2*F*). As with immunofluorescence imaging with individual fragments, we observed again the general pattern of mAb binding to peripheral regions while scFv bound more proximal capsule layers. Co-staining experiments with mAbs 2H1 and 3E5 showed similar patterns, although M4 capsules were bound more weakly (Fig. S4). When single-motif capsules were co-stained with Fab 18B7 and scFv 18B7, scFv consistently outcompeted Fab for capsular binding, although Fab binding was still detectable at longer exposures (Fig. S4).

### mAbs and associated fragments exhibit specificity modulations toward secreted exopolysaccharide material of single motif strains

**Figure 3:**
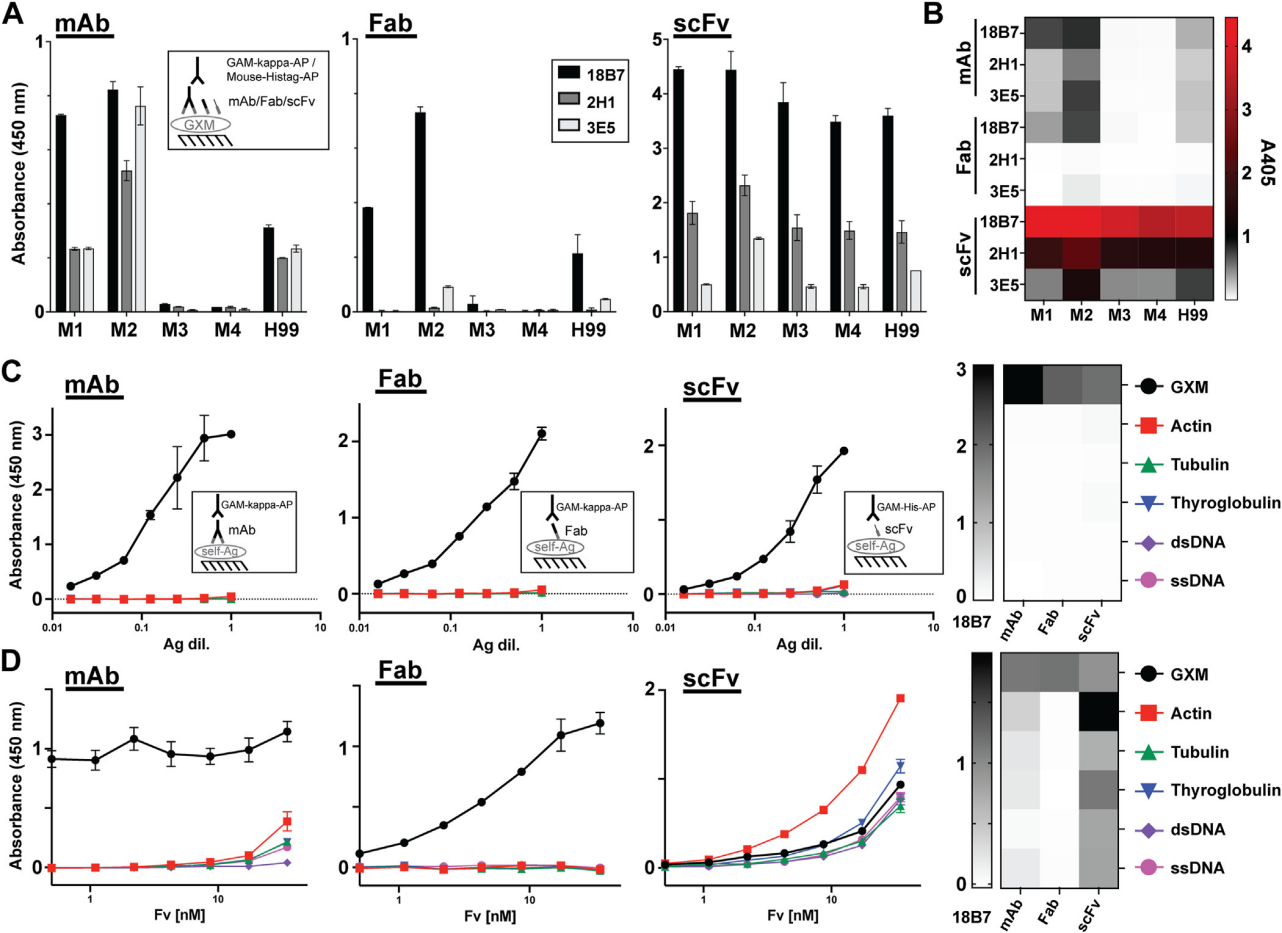
**Specificity of mAbs and associated antibody fragments to single-motif exopolysaccharide and self-antigens.** Summary of indirect ELISA measurements of relative binding of each antibody fragment for 18B7, 2H1, and 3E5 antibody families to exopolysaccharide from four single motif strains and one mixed-motif strain (H99) are presented with respect to antibody fragment class. Each plate was coated with EPS at a concentration of 1 μg/ml. *A*, *left panel*: mAb binding to EPS from different strains at 5 μg/ml ([mAb] = 34 nM, [Fv] = 69 nM). *Middle panel*: Fab binding to EPS from different strains at 3.32 μg/ml ([Fab] = 69 nM, [Fv] = 69 nM). *Right panel*: scFv binding to EPS from different strains at 1.97 μg/ml ([scFv] = 69 nM, [Fv] = 69 nM). Absorbance values for 18B7, 2H1 and 3E5 antibody fragments are displayed as *black*, *gray*, and *silver bars*, respectively. For each fragment classification, binding to EPS isolated from each strain are displayed on the same graph. All A_405_ values are normalized to the values obtained from a control reaction in each experiment on each plate: mAb 18B7 binding to 5 μg/ml H99 EPS. *B*, summary heat map of the data in A–C of each antibody fragment at 69 nM Fv is displayed to illustrate the differences in the magnitude of binding between each fragment class. *C* and *D*, reactivity of mAb, Fab, and scFv 18B7 toward a panel of self-antigens relative to binding to native GXM antigen. Binding profiles of each antibody fragment are displayed in *black*, *red*, *green*, *blue*, *purple* and *pink* for GXM, actin, tubulin, thyroglobulin, double-stranded DNA, and single-strained DNA antigens, respectively. Data represent the mean ± SD of two replicate measurements. Heatmap summaries of the polyspecificity binding data are displayed for the highest concentration of antibody or no dilution of Ag. *C*, antibody concentrations were held constant at 69.4 nM Fv (5 μg/ml mAb) and the antigen was serially diluted two-fold. *D*, antigen concentrations were held constant and antibody concentrations were serially diluted two-fold from an initial concentration of 34.7 nm Fv (2.5 μg/ml mAb).

Given the complexity of motif composition and quaternary structure of capsular GXM as an antigen (41, 42, 43), it is challenging to draw direct conclusions about antibody specificity based on capsule binding. Thus, we examined the contribution of C domains to fine specificity toward to better-characterized exopolysaccharide material that is secreted by single motif-expressing *C. neoformans* strains. All mAbs in this study recognized M1 and M2 exopolysaccharide well, and M3 and M4 exopolysaccharides very weakly. mAb 18B7 bound M1 and M2 exopolysaccharides with roughly equivalent affinity, while mAbs 2H1 and 3E5 exhibited a preference for M2 exopolysaccharide (2.3- and 3.3-fold, respectively). mAbs 18B7 and 2H1 exhibited residual binding to M3 and M4 exopolysaccharides (∼1–2% of binding to M2) but binding by mAb 3E5 was not detectable. The corresponding Fab fragments had similar binding profiles to their parental mAbs but bound with significantly less affinity (Fig. 3, *A* and *B*, and Table S1). Binding was almost completely abrogated for Fabs 2H1 and 3E5, which was predicted given that the affinity of mAb 18B7 is greater than mAb 2H1 (25). The measured affinity of the 3E5 clone was slightly higher than both (25), but IgG3 isotypes are known to aggregate to enhance their functional affinity (44), so the weaker binding of the monovalent Fab 3E5 we observed is not unexpected. The lack of mAb binding to M3 exopolysaccharide contrasted with uniform annular staining observed of all mAbs to all M3 capsules. However, the shared preference of mAbs and Fabs for M2 exopolysaccharide is mirrored in the strong binding of both on the M2 capsules in co-staining experiments, and the poor recognition of M4 exopolysaccharide was consistent with very weak binding of M4 capsules for mAbs 2H1 and 3E5.

Conversely, all scFv fragments exhibited notably broadened reactivity profiles, binding to all motifs tested, most notably at higher scFv concentrations. Each scFv displayed a slight preference for M2 (and M1 to a lesser degree) motifs which was observed more strongly at more dilute concentrations (Fig. S5). scFvs exhibited 4- to 8-fold greater normalized optical densities for exopolysaccharides with M1 and M2 motifs at equivalent 69.4 nM concentrations of Fv, although exact comparisons of relative affinity are not possible due to the different secondary antibodies used for detection (Table S1). The universal recognition of every single motif EPS in this panel by scFv mirrors the strong binding of the corresponding single motif capsules and the general observation that scFvs outcompeted their parental mAbs for M3 and M4 capsules.

### scFv fragments manifest enhanced reactivity toward self-antigens

Given the remarkable expansion of reactivity of scFv fragments for GXM Ags that were not bound by the parental Ab from which they were derived, we explored whether the reactivity expanded further to non-polysaccharide self-antigens. Under normal assay conditions, all fragments exhibited strong specificity for GXM, although some cross-reactivity was observed for scFv 18B7 at the highest Ag concentration (Fig. 3*C*). However, when binding of the mAb 18B7 and its fragment family to a panel of protein and nucleic acid self-antigens was assessed under highly saturation conditions, a significant increase in the relative reactivity of scFv compared to mAb and Fab was observed (Fig. 3*D*). mAb and Fab forms of 18B7 maintained excellent specificity to GXM relative to self-antigens in this panel across the range of antibody concentrations, although minimal off-target binding was detected for mAb at the highest end of the concentration gradient. Conversely, scFv 18B7 recognized all self-antigens in the panel with affinities 75 to 175% that of the native GXM Ag under the saturating Ag conditions (Fig. 3*D*).

### Antibody-mediated glycolysis of the polysaccharide capsule structure is attenuated for fragments devoid of C regions

**Figure 4:**
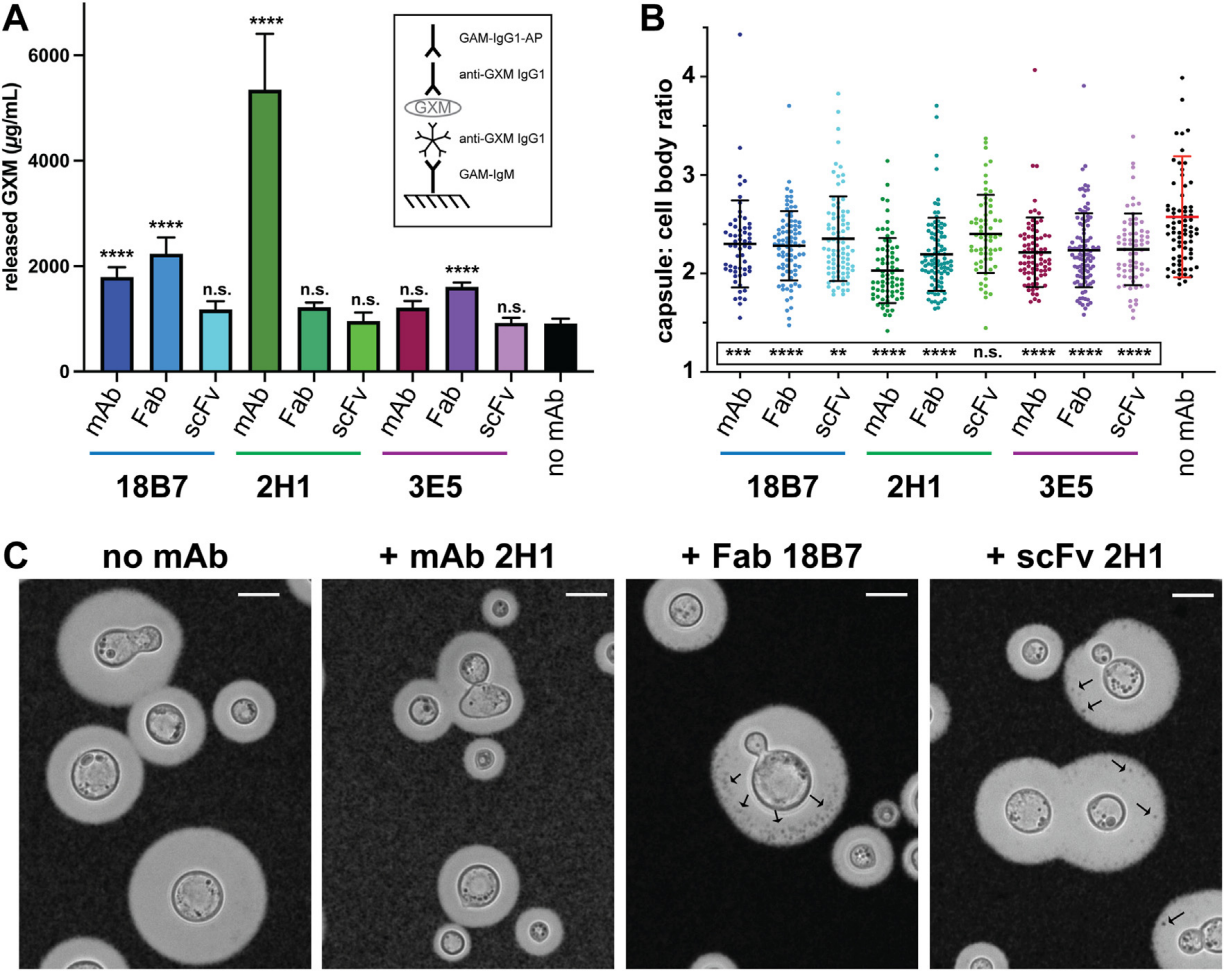
**Glycolytic activity of antibody fragments**. Strain H99 cryptococcal cells were incubated with each antibody fragment for a period of 7 days and catalytic capsule cleavage was assessed. *A*, released capsular GXM concentrations measured by capture ELISA in reaction supernatants after antibody incubation. *B*, the diameters of cell bodies and capsules after antibody incubation were assessed. The ratio of capsule to cell body diameter are displayed for each condition. The mean ratios ± 1 SD are overlaid for each group. Each group contained at least 100 capsule measurements. Statistical significance of released polysaccharide and capsule diameter measurements with respect to ‘no mAb’ controls were determined by ordinary one-way ANOVA (∗∗∗∗*p* < 0.0001; ∗∗∗*p* < 0.001; ∗∗*p* < 0.01; ns, not significant). *C*, representative microscopy images of cryptococcal cells in India Ink after incubation in PBS for 7 days alone or with mAb 2H1, Fab 18B7 and scFv 2H1. *Arrows* indicate the appearance of dark spots inside the capsules of *C. neoformans* cells incubated with Ab indicating India Ink penetrance into damaged regions of capsule. *White scale bars* on all microscopy images indicate 10 μm.

Having established differences in the capsule penetrance and specificity profiles of each antibody fragment, we investigated whether these attributes could result in functional differences. Previous studies have demonstrated that the GXM-specific mAbs in this study can catalytically hydrolyze GXM polysaccharide found in cryptococcal capsules and secreted exopolysaccharide (45, 46, 47), so we examined the catalytic function of these antibody fragments. Heat-killed strain H99 *C. neoformans* cells were incubated with mAb, Fab or scFv forms of 18B7, 2H1, or 3E5 and capsular PS cleavage was quantified indirectly. The concentration of GXM liberated from the capsule into the soluble supernatant and the reduction of capsular diameter were measured as proxies for catalytic activity. In agreement with previous direct measurements of the glycolysis of an M2-type synthetic glycan, mAb 2H1 exhibited the highest degree of glycolytic activity of capsular material (46). MAbs 18B7 and 3E5 hydrolyzed 2.2- and 2.7-fold less capsule than mAb 2H1, respectively. Fab fragments generally hydrolyzed slightly more efficiently than their corresponding mAb, aside from the 2H1 family. Conversely, despite the enhanced and broadened Ag binding by scFvs, the polysaccharide released after incubation with scFv was not significantly greater than GXM spontaneously released from cryptococcal cells in control reactions (Fig. 4*A*). The average diameter of capsules after each antibody incubation was inversely correlated with the amount of released polysaccharide, implying that GXM material sheared from the outer layers of the capsule was the origin of released polysaccharide in the supernatant (Fig. 4*B*). In cryptococcal cells incubated with scFv and Fab fragments, we observed the emergence of dark spots within the halo of the capsule, whereas no such phenomenon was observed in mAb-treated or control cells. This suggests that antibody-mediated damage at the inner regions of the capsule enabled India Ink incursions (Fig. 4*C*).

### Simulations reveal differences in the configuration and dynamics of the paratope in the absence of C domain stabilization

**Figure 5:**
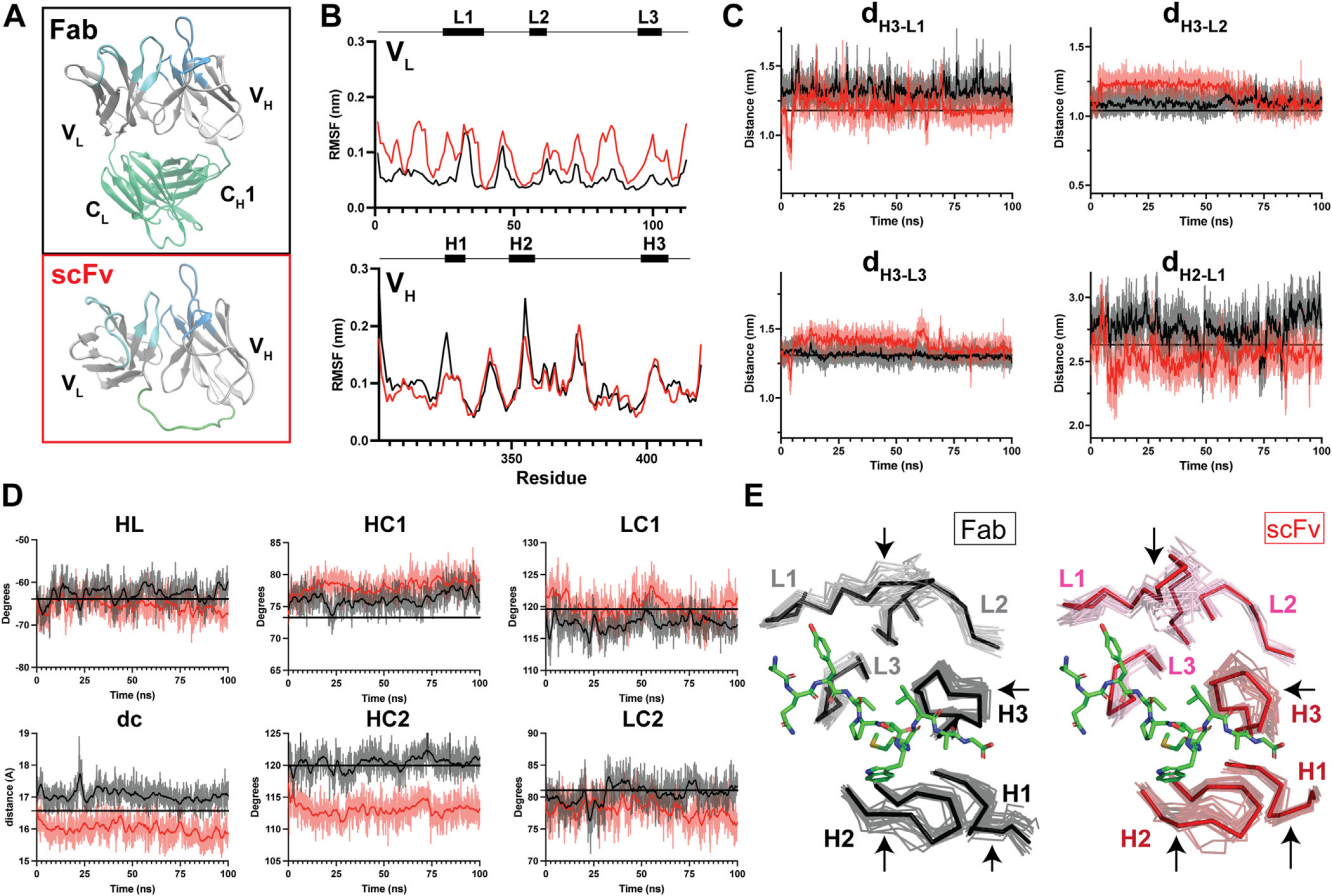
**Molecular dynamics simulations reveal enhanced paratope plasticity of scFv relative to Fab.** 100 ns all-atom molecular dynamics simulations of scFv and Fab 2H1 fragments. Data corresponding to Fab and scFv are presented in *black* and *red*, respectively. *A*, models of the scFv and Fab structures simulated by molecular dynamics. *B*, root mean square fluctuation (RMSF) was calculated for Cα positions of residue in the variable domain of each Ab fragment. The RMSF values are plotted by residue for the V_L_ and V_H_ domains in the *top* and *bottom panels*, respectively. *C*, distances between selected CDR pairs with large variations in distances between Fab and scFv fragments over the respective trajectories. Distances measured from the crystal structure are superimposed as a *black line*. See Fig. S6 for complete distance measurements and the definitions of CDR distances. *D*, orientation of variable domains with respect to one another as defined by ABangle torsion angles (HL), bend angles (HC1, HC2, LC1, LC2) and distance (dc) between variable domains. The calculated ABangle values of the coordinates from 2H1P crystal structure are overlaid as a *black line* for each measure. For both CDR distances and ABangle measurements, the raw data was smoothed with a zero-order polynomial and superimposed on the raw data to reveal general trends. *E*, conformations of scFv (V_L_, *pink*; V_H_, *dark red*; *right*) and Fab (V_L_, *gray*; V_H_, *black*; *right*) were extracted from each trajectory every 5 ns to illustrate the range of conformations sampled by each. The V_H_ domains were then aligned with to the crystal structure and the backbone trace of the CDR conformations along the trajectory are displayed to highlight conformational differences. For each, the conformation of the most populated cluster is superimposed with partially transparent traces of lower-populated states. The peptide mimetic antigen from the co-crystal structure (2H1P) is displayed in *green stick* representation to show the putative Ag binding site. *Arrows* indicate CDRs where significant conformational differences are observed between Fab and scFv.

Having experimentally demonstrated the striking modulation in Ag binding and functional activity between antibody fragments containing C regions and the variable-only scFv fragment, we sought to elucidate the structural basis underpinning these functional differences using molecular dynamics. Because the expansion of Ag recognition observed for scFvs indicated a more plastic paratope, we were especially interested in interdomain motions in the V region. Using the experimentally determined structure of mAb 2H1 (48) as the starting coordinates, 100 ns all-atom molecular dynamics simulations in an explicit solvent were carried out for Fab and scFv fragments. The coordinates of the Fab were fully described by the crystal structure except the C-terminal Cys that forms the interdomain disulfide linkage, which was built into our model. For the scFv, the C domains were removed, and the 15-residue linker was built to join the 2 V domains into a single polypeptide chain (Fig. 5*A*). The paratope is defined by CDR configuration, so we first examined the relative positions of CDR loops during the simulation. The root-mean-square fluctuation (RMSF) of Cα positions in the V regions was calculated, revealing an increase relative to Fab in conformational flexibility for scFv within V_L_ at all CDRs and several framework loops. Interestingly, RMSF values were roughly equivalent for V_H_, with slightly more mobility for Fab in CDR-H1 and -H2 (Fig. 5*B*). Distances measured between CDRs remained relatively stable throughout the 100 ns simulation for the Fab, indicating a well-defined paratope. While the scFv sampled configurations with the same CDR distances as Fab, it also manifested many deviations from the Fab configuration, which were especially evident from distances measured from CDR-H3 to the light chain CDRs. Conformational shifts in scFv yielded primarily changes in interdomain CDR distances, although some intradomain distance modulations (H1-H3, H2-H3, L1-L3) were also observed (Figs. 5*C* and S6).

The relative domain orientations of V domains are another important factor in determining the paratope. V_H_-V_L_ interface and elbow angle fluctuations were previously observed on the low nanosecond timescale (49), while CDR rearrangements are several orders of magnitude slower (50). Thus, we calculated six measures of the V region relationship using the ABangle tool (51) across the simulation trajectory. The average HC1 and LC1 “tilting” bend angles were both slightly higher for scFv through the trajectories. HC1 measures for both scFv and Fab increased a few degrees from the reference crystal configuration. LC1 bend angles remained closer to the reference values but exhibited greater fluctuations. HC2 and LC2 “twisting” bend angles in the scFv simulation exhibited more extreme excursions from the starting configuration than for the Fab. The torsion angle (HL) between a central axis connecting V_H_ and V_L_ was relatively stable and consistent throughout each trajectory, although the scFv trended slowly to smaller angles throughout the simulation (Fig. 5*D*). We also observed that the distance between the reference planes of each variable domain (dc) was noticeably shorter for scFv, as previously reported (52), and there more rapid fluctuations. This comparison of V domain orientation during the simulations suggests that variable domains in the scFv format quickly move ∼1 Å closer than in the Fab and tend to fluctuate away from the starting configurations through tilting and twisting motions. The Fab experienced smaller fluctuations and maintained orientations more similar to the reference structure. To illustrate the range of configurations sampled by Fab and scFv, structures were extracted at every 5 ns along each simulation trajectory and aligned the crystal structure (Fig. 5*E*). CDR loops sampled alternative conformations throughout the trajectory, but this effect was most pronounced for scFv, particularly CDR-L1, CDR-H2, and CDR-H3, whereas those CDRs in the Fab assumed more stable orientations.

Based on the functional differences in glycosidic cleavage observed between the fragments, we also measured the stability of the conformations of putative catalytic residues in the paratope. Many glycosidases feature a pair of residues with acidic side chains (53), and two such potential pairs were identified on the 2H1 paratope (Asp1^VL^-Asp61^VH^ and Asp96^VH^-Asp101^VH^). For both pairs, greater mobility was observed in scFv compared to Fab. In the latter pair, the Asp96^VH^ side chain rapidly assumed an alternative configuration that created an additional 3 Å of separation between the pair (Fig. S7, *B* and *C*). In the previously identified serine protease site (45), the arrangement of the catalytic triad was remarkably stable in the simulation of the Fab while greater fluctuations were measured in the scFv, driven primarily by Ser26^VL^ in CDR-L1 (Fig. S7*A*). In each case, the relative stability of these residues in the Fab could promote greater reactivity of these active sites as compared to the scFv format.

## Discussion

Here we demonstrate that the specificity of three mAbs targeting to cryptococcal capsule relied strongly on contributions from the C regions of the Fab. This study establishes a link between the removal of C regions with paratope flexibility and broadened specificity in this set of mAbs. When combined with the observations of specificity modulation observed in isotype-switched antibodies (11, 12, 16, 47, 54, 55, 56), where V and C_L_ domains are identical, the role of C_H_1 in shaping the paratope was revealed. Sequences of three protective GXM-specific mAbs were selected to generate antibody fragments with varying constant domain content for this study. We first demonstrated that recognition of conserved GXM epitopes was maintained in recombinant scFv 18B7 by demonstrating competition for the binding mixed-motif exopolysaccharide with its mAb and Fab. As expected, based on its bivalency, the mAb was the superior binder. However, when the competition of monovalent 18B7 paratopes was examined, the scFv was superior to the Fab. To assess specificity differences between the fragment families, we analyzed binding to capsular and secreted exopolysaccharides from single motif strains. The binding patterns of the mAb and Fab fragments to single-motif exopolysaccharide demonstrated a strong conserved preference for Motifs 1 and 2. In isolation, all mAbs bound in uniform annular patterns to every single motif capsule tested except for Motif 4, which was weakly recognized by mAb 18B7 and exhibited punctate binding patterns when bound by mAbs 2H1 and 3E5. Interestingly, M3 capsular PS was bound well by all mAbs and Fabs, while the corresponding exopolysaccharide was not. This could reflect the availability of additional epitopes within the complex capsule structures of single motif strains despite the identification of only one motif by NMR chemotyping analysis (20). Given past evidence that some GXM-specific mAbs recognize conformational epitopes that are obscured by certain binding assays (27), it could also reflect a difference in the antigenicity of the same material in different assays. Due to the complexity of capsular antigen, we focus our epitope mapping conclusions instead on the binding of well-characterized exopolysaccharide material. In both immunofluorescence and ELISA binding experiments, we observed conserved specificity but reduced binding strength for Fabs relative to their corresponding mAb, presumably due to the loss of avidity. Avidity is especially pronounced in mAbs that bind to repetitive carbohydrate polymers: IgG2 and IgG3 subclasses in humans and mice, respectively, are common in Abs directed to glycan Ags because they can undergo Fc-mediated aggregation to boost intrinsically weak monovalent binding affinities (6, 57, 58, 59).

Binding profiles of scFv fragments to single-motif exopolysaccharides and capsules revealed that the fine specificity in this set of mAbs was heavily influenced by C domains. Despite sharing similar specificity profiles to their parental mAb in dilute conditions, scFvs bound robustly to exopolysaccharide from every motif tested under saturating conditions and exhibited strong uniform binding patterns on all single-motif capsules. In co-staining experiments with parental antibody and corresponding scFv, M2-expressing capsules were generally stained with equivalent intensity, in agreement with the universal recognition of EPS of that motif. Co-staining of M1 and M4 capsules often exhibited single cells bound exclusively by either one antibody fragment or the other, or preferential staining of different faces of the same capsule. These binding patterns suggest cell-to-cell antigenic variability within a single strain, where some pockets of the capsule are recognized by mAb, and the remaining surface is covered by broadly specific scFvs. ScFv completely outcompeted the parental mAb for binding to M3 capsules, suggesting very little availability of mAb-reactive Ag. A further possibility is that antibody binding of the capsule may induce alterations in the capsule structure that could modulate the availability of different epitopes in dual-staining experiments (41). scFvs also bound more proximally to the cell body than mAbs and Fabs, likely because the smaller hydrodynamic radii permitted deeper diffusion into the capsule (60). The cryptococcal capsule is known to increase in density in regions more proximal to the cell body, forming a “molecular sieve” that can sterically occlude the larger mAbs (61, 62). Alternately or concomitantly, since antibodies are known to penetrate the *C. neoformans* capsule to reach the cell wall (63), it is possible that the differential specificity of scFvs caused preferential binding to the inner aspects of the capsule.

As the antibodies in this study were previously shown to catalyze the hydrolysis of their glycan Ag, we also assessed the relative catalytic activity of each antibody with respect to each fragment. For mAbs 18B7 and 3E5, their corresponding Fabs were more efficient catalysts, likely due to enhanced diffusion properties which enable additional contact with less accessible GXM substrate within the capsule. The relatively lower affinity of Fabs may also result in the release of more catalytic products into the supernatant for downstream detection. As previously reported for a synthetic GXM substrate (46), we observed that mAb 2H1 hydrolyzed GXM more efficiently than any antibody fragment in our panel, including its own Fab. This greater activity could reflect differences in the mAb 2H1 paratope relative to that of mAbs 18B7 and 3E5, which may attenuate catalytic activity when not constrained by the Fc. Despite the broader binding of scFvs, they were relatively inert in the activity assays. From a catalytic perspective, the higher apparent affinity of scFvs may hamper cleavage due to the slower release of the end-product and thus slower catalytic turnover. Alternatively, the conformational restrictions imposed by C domains that define the specificity of mAbs and Fab fragments enforce the correct geometry of the Fv dimer to form the appropriate catalytic center responsible for this reaction, as substantiated by the relative mobility of putative catalytic residue configurations in the scFv format in our dynamics simulations. This phenomenon may also be a consequence of local effects: The deeper average capsular penetrance of scFvs places them in capsule zones that are highly crosslinked and thus more resistant to catalysis. Further, the release of cleavage products into the supernatant may be inhibited by the network of stabilizing interactions in denser capsule layers that retain the fractured polysaccharide in the capsular matrix. This notion is supported by the appearance of dark pockets within the capsule during India Ink staining after incubation with Fabs and scFvs, but not mAbs. This suggests that cleavage of capsular material by smaller Ab fragments, which can occur primarily in the inner layers, weakens the exclusionary properties of the capsule to India Ink dye, but may not result in significant release of PS.

The relative orientation of V domains is the major factor beyond CDR composition in determining Ab specificity. Framework region residues that are located at V_H_-V_L_ interface are more likely to be mutated during somatic hypermutation than other framework residues, suggesting the importance of tuning the interface to achieve the optimal conformation (64, 65). The elbow angle defines the bend between V and C regions in the Fab and is restricted to a specific range, anchored by a “molecular ball-and-socket” joint located at the interface between V_H_ and C_H_1 (3). This relationship helps define the shape of the paratope but in the scFv format, no constraints on the orientation of the V_H_-V_L_ interface are imposed apart from the dimerization interface and the length of the linker connecting the two domains. Although scFvs have been demonstrated to bind the same Ag in a highly similar manner to the corresponding Fab (66, 67, 68), Ab-Ag interactions are generally understood to be driven by inherent plasticity in the binding site in the protein which can shift to accommodate binding (69, 70, 71). Abs are thought to exist in a conformational equilibrium such that binding to a particular Ag is dependent on conformational exchange into an appropriate binding state after Ag encounter (72).

Based on our molecular dynamics analysis, the V region undergoes significant dynamic fluctuations on the low nanosecond timescale when unconstrained by the C regions. The dynamics observed were dominated by rigid-body motions of the V domains with respect to one another, producing a wider range of paratope configurations and unlocking broader Ag recognition. V domain reorientation, especially in the absence of C region stabilization, is relatively rapid due to the low kinetic barrier of nonspecific nonpolar interactions, compared to the high energy barriers of loop rearrangements that are governed by breaking of specific hydrogen bond or electrostatic networks in polar environments (73). The removal of the stabilizing interface between the V and C domains, which imposes restricted configurational relationships on the V domains, permits enhanced exchange of the scFv paratope. Further, the covalent tethering points in the Fab, which join the C-terminal end of the V domain to the N-terminal region of the adjoining C domain are replaced by an engineered linker that joins the C-terminus of V_L_ to the N-terminus of V_H_. Not only are these covalent restraints placed differently, but the connections in the Fab are highly static as part of a structured domain, while the unstructured linker would allow considerable conformational freedom. The remarkable expansion of reactivity observed for scFv formats suggests that a more plastic paratope can accommodate different Ags.

Antibody paratopes are large interfaces, composed of ∼50 CDR residues. However, only a subset of those residues are involved in binding the specific target (74), which theoretically allows unused CDR residues to contribute to binding events of secondary antigens when favorable stereochemical opportunities arise. When the paratope is stabilized by constant domains, antibodies exhibit remarkable selectivity to their primary target, although cross-reactivity is not uncommon (75, 76, 77). Low-affinity cross-reactions with secondary antigens can be facilitated more easily with enhanced paratope flexibility, as demonstrated here for the scFv format. Given that a ten-fold difference in antibody dissociation constant equates to only 5.7 kJ/mol of free energy, the unique mobility of the scFv paratope may allow for the formation a single additional hydrogen bond (free energies of 2–8 kJ/mol) with a secondary antigen which could feasibly raise the affinity to the level of a detectable binding (78), whereas the same paratope tethered to C domains cannot. However, it should be noted that scFvs in our study retain similar specificity to their parental mAb under dilute conditions. It is only under saturating conditions, where the probability of encounter with secondary antigens is higher, that the effect of the unstabilized paratope on polyspecificity is observed. Nevertheless, given the diversity of structure and function and immunoglobulins, one should avoid extrapolating the effects described here to other paratopes. Furthermore, we acknowledge the possible contributions of engineered linkers and epitope tags on scFv constructs employed in this study to the observed polyspecificity.

There is already considerable evidence for antibody isotype affecting specificity (12, 54, 55, 56, 79, 80). Isotype-switched antibodies with different heavy-chain C domains yield altered specificity, and it has been suggested that the altered V_H_-C_H_1 interface may impose new relative orientations of the V_H_-V_L_ interface which could re-shape relative CDR orientations and modulate the specificity of the resultant paratope (11, 14, 47). Mechanistically, the transmission of allosteric information between V regions and C_H_1 of different isotypes has been reported in several isotype-switching studies (reviewed in (16)), and a specific network of closely interacting amino acids at the V_H_-C_H_1 interface has been proposed to serve as a conduit to propagate structural changes from C to V domains (81). ScFvs in our study recognized a broad set of GXM antigenic variants, and this polyreactivity extended further to a panel of self-antigens ranging from proteins to nucleic acids. Conversely, the corresponding mAb and Fab fragments retained a narrow specificity to GXM. Polyreactive antibodies bind a variety of unrelated Ags by a mechanism that is normally thought to derive from an excessively flexible Ag binding surface, resulting in a wide range of nonspecific interactions of low affinity (82). It is estimated that half of IgM molecules are polyreactive, providing wide Ag coverage at lower affinity (82). One possible explanation is that IgMs are promiscuous binders due to the relatively weak V_H_-C_H_1 interactions that allow greater paratope flexibility, which are subsequently replaced with more static interfaces after the substitutions with IgG C_H_1. Accordingly, rigidification at this interface after affinity maturation has been demonstrated by molecular dynamics (49).

The only known natural examples of antibody molecules devoid of C_H_1 domains are homodimeric heavy-chain mAbs. In sharks, the new antigen receptor (IgNAR) is the archetype of this ancient antibody molecule, consisting of only heavy chains. Cartilaginous fish are the most ancient vertebrates known to possess adaptive immune responses with humoral components, and thus provide a window in the antibody evolution (83, 84, 85). Camelid HCAbs are another example of antibody molecules lacking light chains; however, this antibody type appears to have arisen more recently from a single nucleotide polymorphism which resulted in the excision of C_H_1 during mRNA splicing (86). In both examples, a single V domain defines the paratope and is directly adjacent to the hinge region, as opposed to the analogous four-domain Fab arm of conventional Abs. Because the paratope of HC-only antibodies is defined by a single domain, stabilization by additional C domains N-terminal to the hinge region is apparently not required. Conversely, conventional antibodies with V domain heterodimer paratopes require C region scaffolding in the Fab region to lock the relative orientation of the V domains and maintain the fidelity of the paratope. As demonstrated in this study, the removal of C_H_1 and C_L_ domains from the Fab hampers fine-tuned Ag discrimination. Indeed, the promiscuous binding of scFv fragments to self-antigens could have potentially disastrous autoimmune effects and may explain why there are no reported isotypes of antibodies with dimeric Fvs that lack C_H_1 domains. It seems plausible that the evolutionary tradeoff of using a pair of V domains to generate a more complex epitope footprint was the requirement for C_H_1-mediated stabilization of the dimeric Fv. These results suggest that isotype and class switching, previously relegated to the domain of effector function, should be recognized as important determinants for antibody specificity, and carry fundamental implications for our understanding of antibody specificity and the design of recombinant antibody modalities lacking C regions.

## Experimental procedure

### Preparation of Fab fragments

Fab fragments were generated through the treatment of mAbs 18B7, 2H1, or 3E5 with immobilized papain and purification by Protein A-conjugated Sepharose, using the Fab Preparation Kit (Thermo Scientific, 44985) and following manufacturer’s instructions. The purity of Fab fragments was assessed by SDS-PAGE and concentrations were determined by A_280_.

### ScFv plasmid construction

The plasmids for scFv expression were constructed by Gibson Assembly. The nucleotide sequence for each scFv was designed in the V_L_-linker-V_H_ format using the published primary sequences of 18B7, 2H1, and 3E5 V regions. A 15 amino acid (GGS)_5_ linker was engineered between domains and a FLAG epitope tag was appended at the C-terminus of the V_H_ domain. The nucleotide sequences were codon optimized for bacterial expression and synthesized as gBlocks (Integrated DNA Technologies). The pET22b+ expression vector was linearized by PCR with Phusion High Fidelity polymerase (New England BioLabs, M0530L) and primers to add adapter sequences to the 5′ and 3′ ends for the Gibson Assembly reaction. The expression vectors were then assembled using the Gibson Assembly Master Mix (New England BioLabs, M5510A). Assembly reactions were then transformed to Subcloning Efficiency DH5α competent cells (Invitrogen, 18265-017) and transformants were selected on LB-Agar plates supplemented with 100 μg/ml ampicillin. Single colonies were then expanded and sequenced confirmed by Sanger sequencing.

### ScFv expression and purification

Plasmids encoding each scFv construct (pET22b+, 69744-3, Novagen) were transformed to BL21 STAR *Escherichia coli* cells for protein production. A 5 ml volume starter cultures were inoculated from a single transformant colony on LB Agar + Amp plates. After overnight growth, starter cultures were used to inoculate 500 ml expression cultures in ZYP-5052 autoinduction media (1% tryptone, 0.5% yeast extract, 10 g NaCl, 50 mM Na_2_HPO_4_, 50 mM KH_2_PO_4_, 25 mM (NH_4_)_2_SO_4_, 0.5% glycerol, 0.05% glucose, 0.2% alpha-lactose, 2 mM MgSO_4_, 200 μl trace elements (Teknova, Cat no. T1001)). Cultures were grown until OD_600_∼0.6, then the temperature was dropped to 18 °C for overnight expression. Cultures were then spun down 4000*g* for 15 min at 4 °C to obtain the cell pellet for further processing.

#### For periplasmic extraction

Cell pellets were resuspended in 20% sucrose, 1 mM EDTA, 30 mM Tris-HCl, pH 8.0 at room temperature, using 80 ml/g wet pellet. The suspension was shaken gently at room temperature for 10 min. Cells were then pelleted at 13,000*g*, 4 °C for 10 min. The supernatant was removed carefully, and the pellet was rapidly resuspended in the same volume of ice-cold ddH2O and shaken gently at 4 °C, 10 min. The cells were again pelleted at 13,000*g*, 4 °C for 10 min. The supernatant (containing the periplasmic fraction) was then analyzed for protein content. Method adapted from (87).

#### For cytoplasmic extraction

Pellets were first resuspended in 15 ml of 50 mM Na_2_HPO_4_, 300 mM NaCl, 2 mM EDTA and then frozen. The frozen pellets were then supplemented with 0.2 mg/ml lysozyme (Thermo Scientific, 89833) and Protease Inhibitor Cocktail (Sigma, #1873580001) and shaken at 37 °C for 30 min to disrupt the cell membranes. Cells were then subjected to another freeze-thaw cycle (frozen at −20 °C, thawed at 37 °C for 15 min in the presence of 25 μl DNase (Invitrogen, #18047019) + 100 mM MgSO_4_ to reduce viscosity). Cells were then pelleted again, and the supernatant was collected for analysis.

#### For inclusion body preparation

After cytoplasmic and periplasmic extractions, the remaining cell pellet was washed once with 50 mM phosphate, 300 mM NaCl, 10 mM EDTA. The supernatant was removed, and the pellet was then washed twice with 50 mM phosphate, 300 mM NaCl, 10 mM EDTA + 1% Triton 100-X. Between each wash, the insoluble material was pelleted with 10 m centrifugation steps at 15,000*g* and 4 °C. The washed pellets were then resuspended in 100 mM phosphate, 10 mM Tris HCl, 8 M urea, pH 8 overnight at 4 °C with gentle shaking to solubilize the inclusion bodies. Solubilized inclusion bodies were then centrifuged at 14,000*g* for 10 m. Inclusion bodies were then purified by IMAC purification under denaturing conditions (8 M urea supplemented to all IMAC buffers). Elution fractions containing scFv as assessed by SDS-PAGE were then pooled and supplemented with 60 mM GSH to fully reduce all disulfides for 1 h at room temperature. To re-fold the denatured inclusion bodies, the reduced inclusion bodies were then flash-diluted (1:80) in refolding buffer (50 mM Tris-HCl, pH 8.0, 5% glycerol, 0.5 mM GSSG). The refolding was allowed to proceed overnight at 4 °C with gentle stirring. This step is to re-form the disulfides in dilute conditions, such that no intermolecular disulfides will form, only proper intramolecular bonds, while also diluting urea and imidazole. Finally, a second IMAC affinity purification step was conducted. 10 ml of washed IMAC resin slurry was added to the bottle containing the refolded scFv and mixed gently for 10 min. The resin:scFv mixture was then passed slowly through a gravity column and eluted in PBS supplemented with 300 mM imidazole. Elution fractions containing pure scFv were then pooled and the buffer was exchanged for PBS for long-term storage.

### Circular dichroism

18B7 mAb, Fab, and scFv were analyzed by circular dichroism using an Aviv-420 spectropolarimeter (Biomedical Inc). Protein purity was assessed by gel electrophoresis and the concentrations were measured by A_280_ absorbance using molar absorptivity coefficients calculated from the primary sequences. The concentrations of mAb, Fab, and scFv 18B7 for 18B7 were 0.382, 0.555, and 0.174 mg/ml. 400 μl of these protein stocks were loaded into a 1 mm CD cuvette and the CD spectra were acquired at 25 °C from 190 to 260 nm with 1.0 nm wavelength steps, 3.0 s averaging times, and 0.333 s settling times. The intrinsic ellipticities of the buffer controls were subtracted from each experimental sample and the ellipticity was then converted from millidegrees to mean residue ellipticity by the following equation, to adjust for differences in the concentration and composition of each protein fragment:

$$\text{Ellipticity}\;\left(\text{in}\;\deg\;{\text{cm}}^{2}\;{\text{dmol}}^{-1}\right)=\left(\text{millidegrees}\times \text{mean}\;\text{residue}\;\text{weight}\right)/\left(\text{pathlength}\;\left(\text{mm}\right)\times \text{concentration}\;\text{in}\;\text{mg}\;{\text{ml}}^{-1}\right)\text{.}$$

### Size-exclusion chromatography and multi-angle light scattering

mAb, Fab, and scFv 18B7 fragments were all analyzed by SEC-MALS to ascertain the absolute molecular mass of each protein in the solution. 8.1, 11.1, and 12.4 μg, respectively, of each fragment were injected through an Agilent 1200 Series HPLC (Agilent) system equipped with a diode array detector and refractor index detector, coupled to a MiniDAWN multiangle light scattering detector system (WYATT Technology). Antibody fragments were separated on a ThermoScientific Acclaim SEC-300 (300 Å, 5 μm, 4.6 mm × 300 mm) using a flow rate of 0.3 ml/min and modified DPBS (Cytiva; SH30028.02). The absolute molar masses were calculated using Astra 8.1.

### *C*. *neoformans* cell culture

Cryptococcal cultures in 5 ml YPD media were inoculated from cell stocks maintained at −80 °C and grown to confluence for 2 days 1 ml of YPD starter culture was then used to inoculate 100 ml of CN Minimal Media (30 mM KH_2_PO_4_, 10 mM MgSO_4_, 13 mM glycine, 16.7 mM glucose, 6 μM thiamine, pH 5.5) to induce capsule enlargement and grown for 3 days. Capsule-induced cryptococcal cells were when centrifuged for 10 min, 4000*g* and the supernatant was removed. The cells were then washed 3 times in 1× PBS to remove excess exopolysaccharide. Cells were then heat-killed with a 30 min, 65 °C heating step. Loss of cell viability was confirmed by plating these cells on YPD Agar plates and confirming no colony formation after a 3-day incubation at 30 °C.

### Cell-based antibody-mediated *C. neoformans* capsule degradation assays

Heat-killed CN cells were diluted to 5E7 cells/ml in PBS to a final volume 500 μl and supplemented with 50 ug/ml mAb (equivalent to 0.4 uM Fv). The cryptococcal cells were incubated with mAb, Fab or scFv for 7 days at 37 °C. After incubation, the cells were pelleted at 4000*g* for 10 min and the supernatant was collected for analysis. The supernatant was then passed through a 0.22 μm filter (Covidien 1 ml syringe sterile (8881501400); Monoject Tuberculin Syringes (Fisher Scientific 22-257-154)) to remove residual cells. Cells after antibody treatment were washed 3× in ddH_2_O and mixed with India Ink for capsule sizing measurements.

### Enzyme-linked immunosorbent assays

All incubations are carried out at 50 μl volumes for 1 h at 37 °C with gentle shaking unless otherwise indicated. All wash steps involve 3× washes with Tris-buffered Saline + Tween-20 (TBST; 10 mM Tris-HCl, 150 mM NaCl, 1 mM sodium azide, 0.1% TWEEN-20, pH 7.2) using a MultiWash+ Microplate Washer (Molecular Devices).

GXM exopolysaccharide antigen was harvested from the fungal culture supernatant of the indicated *C. neoformans* strain (multi-motif expressing strain H99 used for standards), which we refer to as exopolysaccharide. Briefly, 5 ml YPD starter cultures were inoculated from frozen stocks and grown at 30 °C for 48 h. The cultures were then expanded into CN Minimal media by inoculation with the YPD culture (1:500) and grown at 30 °C for 72 h. The cells were then pelleted, and the supernatant was passed through a 0.22 μm filter (Millipore, # GSWP29325) to eliminate any remaining cells. The exopolysaccharide samples were then concentrated 100-fold by lyophilization. The concentrations of the exopolysaccharide standards were quantified through dry mass measurements and confirmation by the phenol-sulfuric acid method (88).

#### Indirect ELISA single motif exopolysaccharide specificity

96-well high-binding polystyrene plates (Corning, 9018) were coated with antigen with an initial incubation with 1 μg/ml EPS from single motif strains 24067 (M1), Mu-1 (M2), 409B (M3), KT24066 (M4) and H99 (mixed motif standard), dissolved in Phosphate Buffered Saline (PBS; 137 mM NaCl, 2.7 mM KCl, 1.5 mM KH_2_PO_4_, 8.5 mM Na_2_HPO_4_), and then blocked with 200 μl of blocking solution (1% w/v bovine serum albumin (BSA), 0.1 M sodium azide, dissolved in TBST). The plates were washed and incubated overnight at 4 °C with antibodies or antibody fragments at constant Fv concentrations of 69 nM, corresponding to 5.00, 3.32, and 1.96 μg/ml for mAb, Fab, and scFv, respectively. Plates were washed and incubated with Goat anti-Mouse Kappa-AP (Southern Biotech; 1050-04) or Mouse anti-His-tag-AP (Southern Biotech; 4603-04) for mAb and Fab or scFv, respectively. For detection, plates were incubated with 1 mg/ml of para-nitrophenylphosphate substrate (PNPP; Sigma #P5994) dissolved in PNPP Substrate Buffer (1 mM MgCl_2_ ∗6 H_2_O, 50 mM Na2CO_3_, pH 9.8) and the absorbance at 405 nm was measured in each well with the EMax Plus Microplate reader (Molecular Devices).

#### Capture ELISA for released capsular polysaccharide quantification

Plates were coated with 1 μg/ml unlabeled Goat anti-Mouse IgM (Southern Biotech; 1020-01) dissolved in PBS, then blocked with 200 μl of blocking solution. The plates were then washed and incubated with 2 μg/ml mAb 2D10 IgM (described previously (24)). The plates were washed, and incubated with GXM standards (at concentrations from 5 μg/ml to 78 ng/ml) and reactions with unknown GXM concentrations (each in duplicate, at 1:500 dilutions, serially diluted 3×) overnight at 4 °C. The plates were washed and incubated with 5 μg/ml mAb 18B7 IgG1 (described previously (24)), washed, incubated with 1 μg/ml Goat anti-mouse IgG1-HRP (Southern Biotech; 1070-04) and washed.

For detection, plates were incubated with 1 mg/ml of para-nitrophenylphosphate substrate (PNPP; Sigma #P5994) dissolved in PNPP Substrate Buffer (1 mM MgCl_2_ ∗ 6 H_2_O, 50 mM Na2CO_3_, pH 9.8) and the absorbance at 405 nm was measured in each well with the EMax Plus Microplate reader (Molecular Devices). Absorbances from the unknown samples were interpolated into the standard curve to determine the absolute concentration of GXM.

#### Competition ELISA

Plates were coated with antigen with an initial incubation with 1 μg/ml with *C. neoformans* strain H99 exopolysaccharide and blocked with 200 μl of blocking solution. Plates were washed and either mAb or Fab 18B7 were added. 100 μl of 5 μg/ml (67 nM Fv) competing mAb 18B7 or 3.3 μg/ml Fab 18B7 (67 nM Fv) was added to wells 1A, 1B, 1C, and 1D, and serially two-fold diluted across the columns. To every well in rows A and B, 50 μl of 0.36 μg/ml (13 nM Fv) of detected scFv 18B7 was added, and 50 μl of blocking solution was added to every well in rows C and D (no competition controls). Therefore, in the competition experiments in rows A and B, 40 M equivalents of mAb or Fab to 1 M equivalent of scFv (with respect to Fv) were present in column 1. For detection in both experiments, 50 μl Mouse anti-His-Tag secondary antibody conjugated to alkaline phosphatase (Southern Biotech, 4603-04) was added, incubated, and washed. Plates were then incubated with 1 mg/ml of para-nitrophenylphosphate substrate (PNPP; Sigma #P5994) dissolved in PNPP Substrate Buffer (1 mM MgCl_2_ ∗ 6 H_2_O, 50 mM Na2CO_3_, pH 9.8) and the absorbance at 405 nm was measured in each well with the EMax Plus Microplate reader (Molecular Devices). The ratio of average optical densities from rows A-B:C-D for each column was calculated to give a percentage of binding at each antibody concentration.

#### Polyreactivity assay

Polyreactivity of the 18B7 antibody fragment family was assessed with a set of antigens by ELISA with a protocol adapted from (13). Briefly, ELISA plates were coated with actin (A3653, Sigma), tubulin (T240-A, Cytoskeleton, Inc), and thyroglobulin (T1001, Sigma) at 5 μg/ml in bicarbonate buffer (1 mM MgCl_2_ ∗6 H_2_O, 50 mM Na_2_CO_3_, pH 9.6), and with double-stranded DNA (D8515, Sigma) and single-stranded DNA (D8899, Sigma) at 100 μg/ml in citrate buffer (1.2 mM citric acid, 8.7 mM trisodium citrate dihydrate, pH 6.0), and with 1 μg/ml GXM in PBS, pH 7.0, overnight at 4 °C. In the first experiment, the antigens were serially diluted from those starting concentrations, except the starting concentration of GXM was 5 μg/ml. In the second experiment, the antigen concentrations were constant. In the first experiment, mAb, Fab, or scFv concentrations were held constant at 69 nM of Fv. In the second experiment, plates were then blocked for 2 h at 4 °C, washed, and incubated with mAb, Fab, or scFv 18B7 at 34 nM of Fv (2.5, 1.7, or 0.98 μg/ml, respectively), and serially diluted. Plates were incubated for 1 h at 37 °C, and binding was determined by addition alkaline phosphatase-conjugated secondary antibodies (Goat anti-Mouse IgG1 (1070-04, Southern Biotech), Goat-anti Mouse kappa (1050-04, Southern Biotech), or Mouse anti-His-Tag (4603-04), for mAb, Fab and scFv, respectively).

### Immunofluorescent microscopy of antibody localization on *C. neoformans* capsules

Cryptococcal strains were grown as described above in minimal media for capsule induction. After 3 days of growth in minimal media, cells were pelleted and washed three times in PBS to remove exopolysaccharide. Cells were then diluted to 1 × 10^8^ cryptococcal cells/ml in 100 μl and incubated with GXM-specific primary antibody or antibody fragments normalized 34 nM Fv for 1 h. Cells were washed again three times in PBS to remove unbound antibodies. For staining for individual antibody fragments, cells were then visualized with fluorescent secondary antibodies. ScFv were visualized with Mouse anti-His-Tag-AF488 (Southern Biotech; 4603-30) and mAb and Fab were visualized with Goat anti-Mouse IgG (H+L) TRITC (Southern Biotech, 1031-03). The cells were washed again three times in PBS and mounted on slides with ProLong Gold. Images were collected on Leica THUNDER Live Cell & 3D Confocal Microscope and Olympus IX 70 microscope (Olympus America) equipped with standard FITC and 4′,6-diamidino-2-phenylindole (DAPI) filters. For the measurement of fluorescence intensities to quantify antibody penetrance into the capsule, the RGB Profiles tool in Fiji (89) was utilized. The beginning and end points of antibody staining, and cell body boundaries, were identified as the points in at which the maximum or minimum derivative of the fluorescence intensity plot occurred. For comparison across cells of different dimensions, the capsular binding distances were normalized to the cell body diameter for each individual cell.

### Molecular dynamics simulations

The starting coordinates of the anti-GXM Fab 2H1 were taken from the crystal structure of 2H1 in complex with the P1 peptide mimetic (48) (resolution: 2.40 Å, PDB entry: 2H1P). The coordinates for the peptide antigen and the waters were stripped and 2 C terminal residues were added to C_H_1 (Asp521 and Cys522) using MODELLER (90). Disulfide linkages were added were added to each of the immunoglobulin domains with “gmx pdb2gmx” (Cys23-Cys93 and Cys322-Cys396 for scFv, additionally Cys139-Cys199 and Cys447-Cys502 and the intermolecular Cys219-Cys522 for Fab; 2H1P crystal numbering). The scFv 2H1 structure was modeled from the same crystal coordinates, but V domains were omitted. Thus, only coordinates for Asp1-Lys112 and Asp301-Ser420 (crystal numbering) were retained. The 15-residue (GGS)_3_ utilized in the constructs in this study was built between the C-terminus of V_L_ and the N-terminus of V_H_ using MODELLER (90). Molecular dynamics (MD) simulations were carried out using GROMACS 2023.2 (91) and the all-atom CHARMM36 force field (92). Both Fab and scFv models were then solvated with TIP3P waters and centered in dodecahedron simulation boxes with at least 10 Å between the antibody fragment and box edge. The charge was neutralized with Na^+^ and Cl^−^ ions to a concentration of 150 mM. The assembled solvated systems were then energy minimized with the steepest descent method to yield maximum forces less than 1000 kJ/mol/nm. To equilibrate the solvent system 100 ps simulations were conducted under NVT and NPT ensembles, to stabilize the system at 300 K and atmospheric pressure, respectively. The production MD was carried out for both fragments for 100 ns, with a time step of 2 fs.

### MD trajectory analysis

Backbone root mean square fluctuation of V domains (omitting C domains and linker) during the simulations was calculated using the GROMACS “gmx rmsf” module. Distances between CDR pairs throughout the trajectory were calculated using the “gmx distance” module and defined as the distances between the Cα positions of central residues in each CDR. Conformations in the trajectory were binned into clusters based on their RMSD using the “gmx cluster” module with an RMSD cutoff of 0.15 nm and the gromos clustering algorithm. The representative structure from the most populated cluster was aligned to the crystal structure of mAb 2H1, along with structures extracted from each trajectory every 5 ns using the “gmx dump” tool for analysis. The relative V domain orientations for each fragment at 50 ps snapshots throughout the trajectory were evaluated with the ABangle tool (51). ABangle first projects a plane on the V domains based on the positions of conserved Cα positions in a reference set of antibody coordinates. The tool then describes the orientation of V domains using six measurements: the distance and dihedral angle between planes and two bend angles between each plane. These measurements were calculated for each snapshot in the trajectories and plotted as a function of simulation time.

## Data availability

All data are contained within the manuscript.

## Supporting information

This article contains supporting information (29, 48, 93).

## Conflict of interest

The authors declare that they have no conflicts of interest with the contents of this article.
