# Supplementary material for: Immunoglobulin constant regions provide stabilization to the paratope and enforce epitope specificity
Source: J Biol Chem. 2024 May 18;300(6):107397. doi: 10.1016/j.jbc.2024.107397 (PMC11215335; doi:10.1016/j.jbc.2024.107397)
Supplement: Supplemental Figures S1–S7 and Table S1 [file mmc1.docx]

# Supporting Information associated with:

**Immunoglobulin constant regions provides stabilization to the paratope and enforces epitope specificity.**

Scott A. McConnell^1^, Arturo Casadevall^1^.

^1^Department of Molecular Microbiology and Immunology, Johns Hopkins Bloomberg School of Public Health, 615 North Wolfe Street, Baltimore, MD 21205, USA.


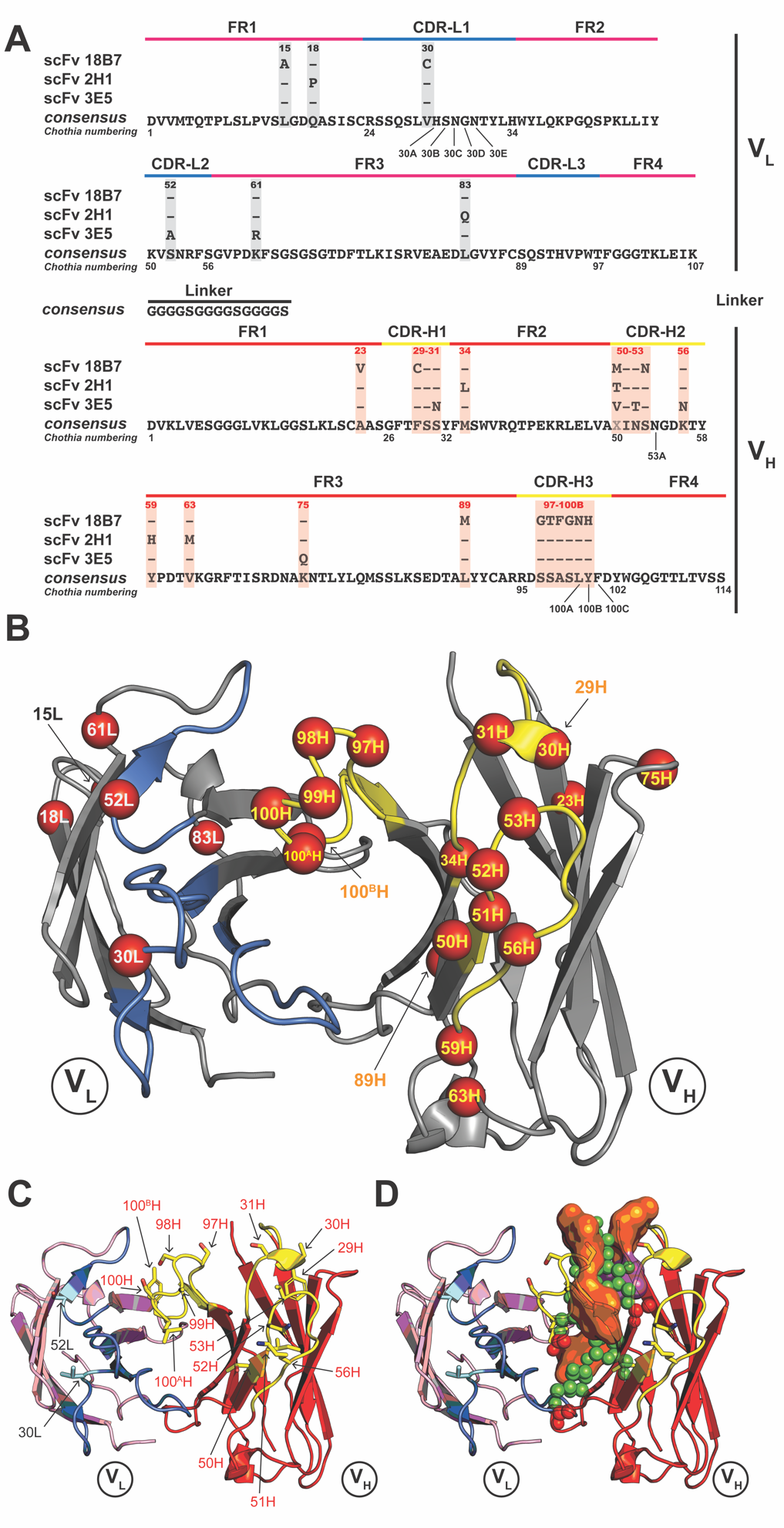


# Supplementary Fig. 1. Sequence and structure alignment of variable regions of mAbs 18B7, 2H1 and 3E5.

Primary and tertiary structural differences between the three protective mAbs characterized in this study are illustrated. **A)** Primary sequence alignment (Chothia numbering) illustrating the differences in amino acid composition between the mAbs. Complementarity determining regions (CDR) and framework regions (FR) are indicated above the primary sequences. At positions with amino acid differences, a dash indicates that the sequence is the same as the consensus sequence, otherwise the substitution is indicated by the one letter amino acid code. **B)** The positions of all amino acid substitutions in the variable domains are visualized on the crystal structure of mAbs 2H1 (PDBID: 2H1P^2^). The variable domains are represented in cartoon representation, with the framework regions, V_L_ CDRs and V_H_ CDRs colored gray, blue and yellow, respectively. The positions with subsitutions are indicated by a red sphere and labeled with their position in Chothia numbering scheme. **C)** Only substitutions located within the CDR loops are indicated on the crystal structure of mAb 2H1. V_L_ and V_L_ domains are distinguished by coloring as pink and red, respectively; CDR loops are colored as before; CDR substitutions are displayed as stick representation of the amino acid at that position in mAb 2H1. **D)** The model of the mAb-GXM Ag complex is displayed to highlight the putative binding cleft (based on model from Crawford, et al^1^). The mannan backbone and acetylation are represented as green and red spheres, respectively. The xylose or glucuronic acids are shown in surface filling representation and colored orange and purple, respectively.


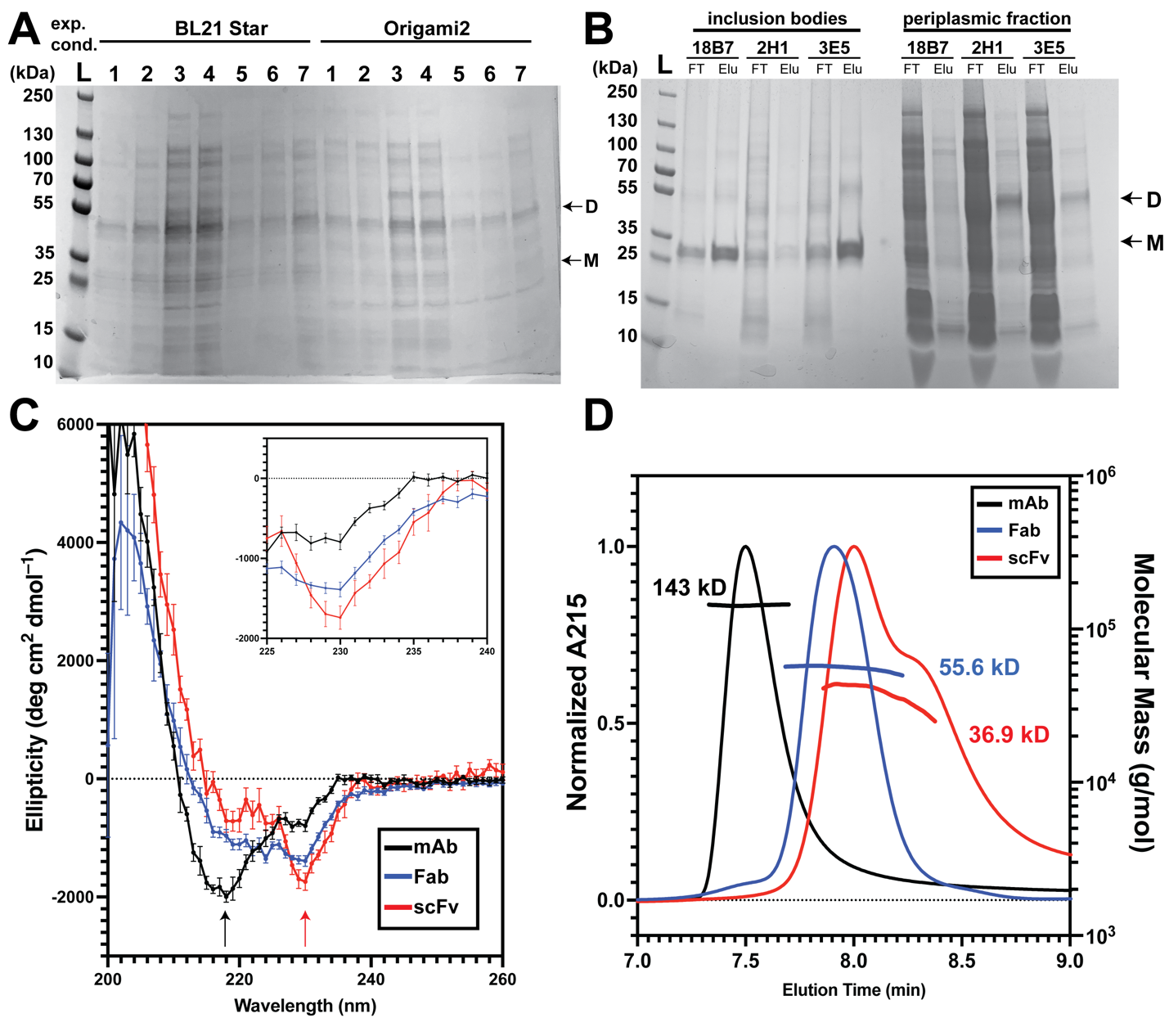


# Supplementary Fig. 2. Optimization of scFv production.

SDS-PAGE analysis of several expression and purification optimization related to scFv production. In each gel image, the bands corresponding to monomeric and dimeric species are indicated with arrows labeled M and D, respectively. **A)** Optimization of periplasmic expression conditions for scFv 18B7 expression. Expression for scFvs 2H1 and 3E5 were similar. For each sample, the periplasmic fraction of bacterial cells was extracted and analyzed on the gel. Seven different expression conditions were tested. Traditional method: Minimal media cultures are inoculated with 1/50 volume overnight precultures grown in Luria Broth. The cultures are induced at OD_600_ = 0.7 and expressed overnight at 17°C. Bracken method: initial growth to OD_600_ = 0.7 in Luria Broth to confluence, then pelleted, washed, and exchanged into ¼ volume minimal media and induced for overnight expression at 17°C. High cell density method: initial growth to OD_600_ = 4 in Luria Broth to confluence, then pelleted, washed, and exchanged into an equal volume of HCD minimal media and induced for overnight expression at 17°C. All conditions were tested for both BL21 Star and Origami2 expression systems, as indicated. Conditions: 1 = Bracken, 0.25 mM IPTG, 2 = Bracken, 0.5 mM IPTG, 3 = HCD, 0.25 mM IPTG, 4 = HCD, 0.5 mM IPTG, 5 = Traditional, 0.25 mM IPTG, 6 = Traditional, 0.5 mM IPTG, 7 = Bracken, 0.5 mM, expressed at 37°C. **B)** Purification of scFv 18B7 from inclusion bodies and the periplasmic fraction. Subsequent flash dilution of denatured inclusion bodies yields monomeric preparations, while periplasmic scFvs are present as a mixture of dimers and monomers. **C)** Far-UV circular dichroism of mAb, Fab and scFv 18B7 suggest that the recombinant scFv has a high degree of beta-strand character and no significant disordered secondary structure. 18B7 mAb, Fab and scFv CD spectra are displayed as black, blue, and red. An expanded view of the region from 200-260 nm is displayed. A negative band at 218 nm expected for antiparallel beta sheets and unique CD band at 230 are indicated by black and red arrows, respectively. Inset) Expanded view of the unique spectral signature of the 18B7 antibody family at 230 nm. **D)** SEC-MALS analysis of the 18B7 fragment family. The chromatogram of UV absorbance at 215 nm and molecular masses for each elution peak are plotted in the same color. The scales UV absorbance and molecular masses are displayed on the left and right axes, respectively. SEC-MALS data corresponding to the different 18B7 fragments are colored as in C). The SEC elution chromatogram indicates a deviation from a Gaussian peak shape, but molecular masses determined by light scattering indicated low polydispersity (+/- 5%). There was no evidence of higher order aggregation.


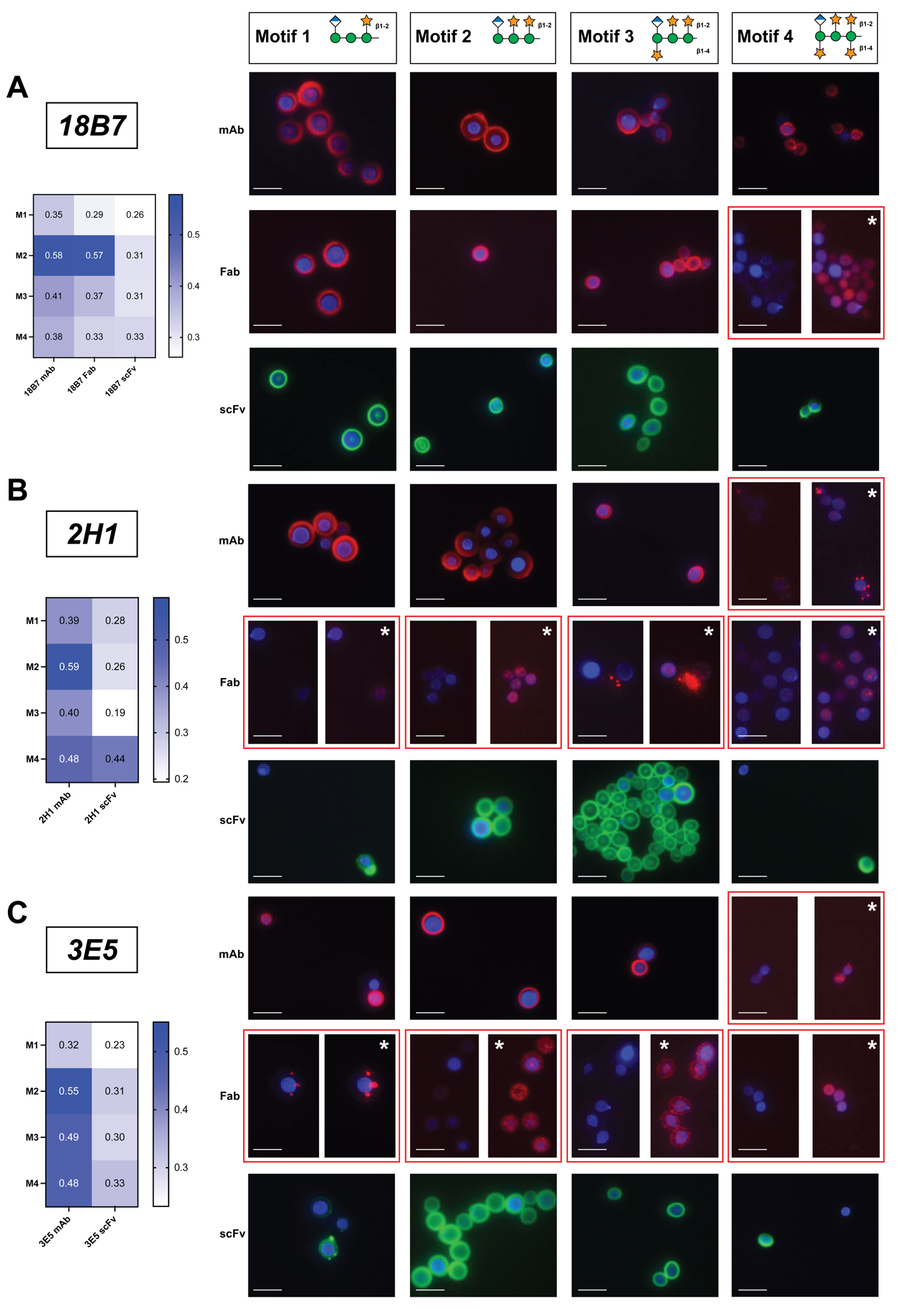


# Supplementary Fig. 3. Capsular binding patterns of mAbs and associated antibody fragments (associated with Fig. 2).

Representative images of antibody staining patterns of each single motif strain (Motifs 1 - 4) are presented for each antibody format. For each motif, the structure of the triad is presented next to the motif. The mannan backbone and xylose and glucuronic acid sidechains are represented as green circles, orange stars and blue-white diamonds, respectively, according to Symbol Nomenclature for Glycans (SNFG). To the left of each family are the average capsule penetrance measurements for each condition. Antibody families 18B7, 2H1 and 3E5 are visualized in panels A, B, and C, respectively. White scale bars on all microscopy images indicate 10 µm. For conditions with relatively weak mAb or Fab binding, the exposure for the red channel was increased to visualize the localization of the mAb or Fab. The images are displayed within a red box to the right of the original image with consistent intensity threshold and indicated with an asterisk (*). The image of the 18B7 mAb binding M2 capsule also appears in the main Figure 2A. For completeness, this images was included in this expanded Supplemental Figure in order to compare the binding of all 18B7, 2H1, and 3E5 (mAb, Fab, and scFv) to the four single motif strains used in this study.


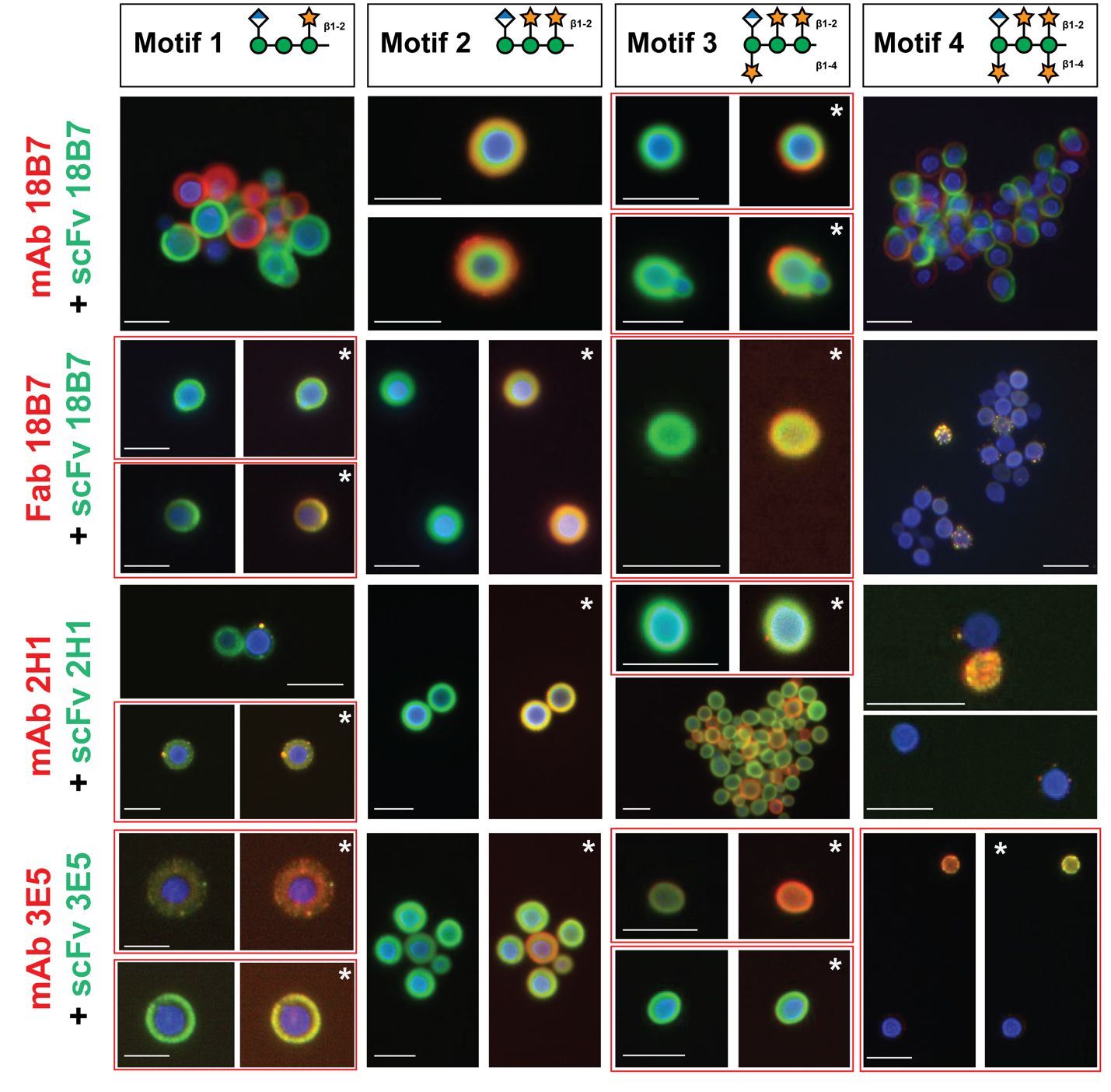


# Supplementary Fig. 4: Capsular binding co-staining patterns of mAbs versus scFvs on single GXM motif expressing strains (associated with Fig. 3).

Representative images after dual staining of all parental mAbs with their corresponding scFv fragments. For each motif, the structure of the triad is presented next to the motif. The mannan backbone and xylose and glucuronic acid sidechains are represented as green circles, orange stars and blue-white diamonds, respectively, according to Symbol Nomenclature for Glycans (SNFG). mAbs and Fabs were visualized with an anti-mouse kappa chain secondary antibody conjugated to TRITC (red) fluorophore and scFvs are visualized with an anti-His tag secondary antibody conjugated to a FITC fluorophore (green). The white scale marker on each image indicates 10 µm. Multiple fields of the slide are shown in top and bottom panels for conditions with few cells in each field. For conditions with relatively weak mAb or Fab binding, the exposure for the red channel was increased to visualize the localization of the mAb or Fab. The images are displayed within a red box to the right of the original image with consistent intensity threshold and indicated with an asterisk (*). The images of mAb 18B7/scFv 18B7 binding M1 capsules, M2 capsules (bottom), and M4 capsules also appear in the main Figure 2D, 2E, and 2G, respectively. For completeness, these images were included in this expanded Supplemental Figure in order to compare competitive binding between corresponding mAbs and scFvs/Fabs for all three mAbs in this study.


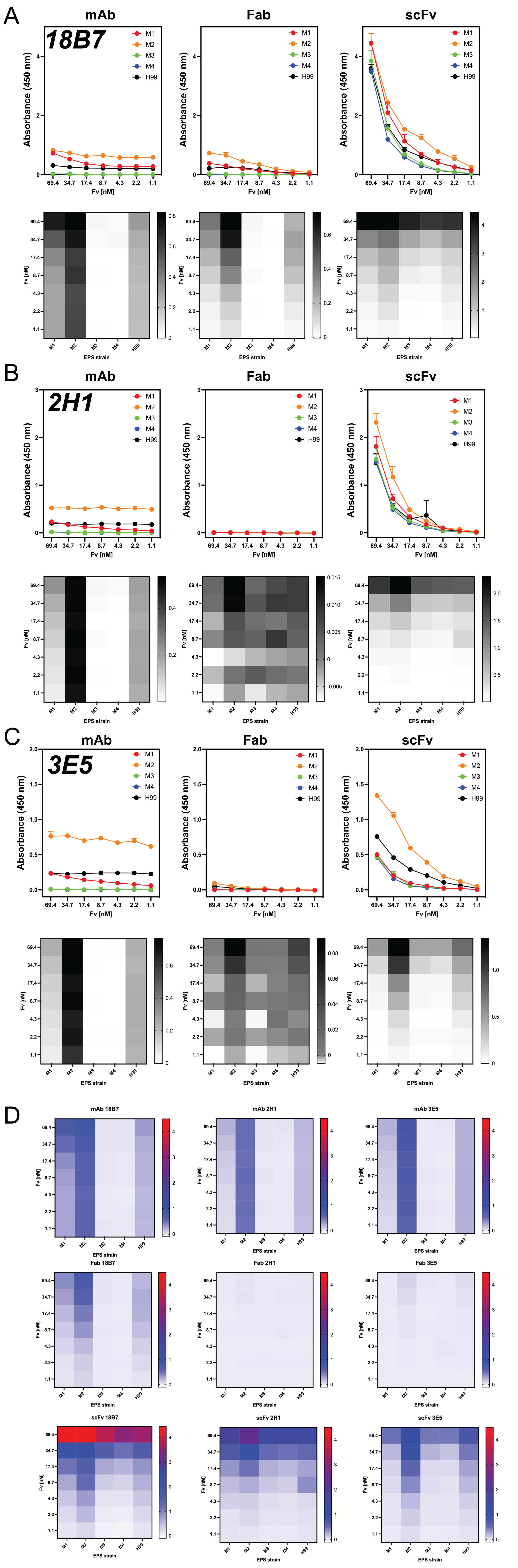

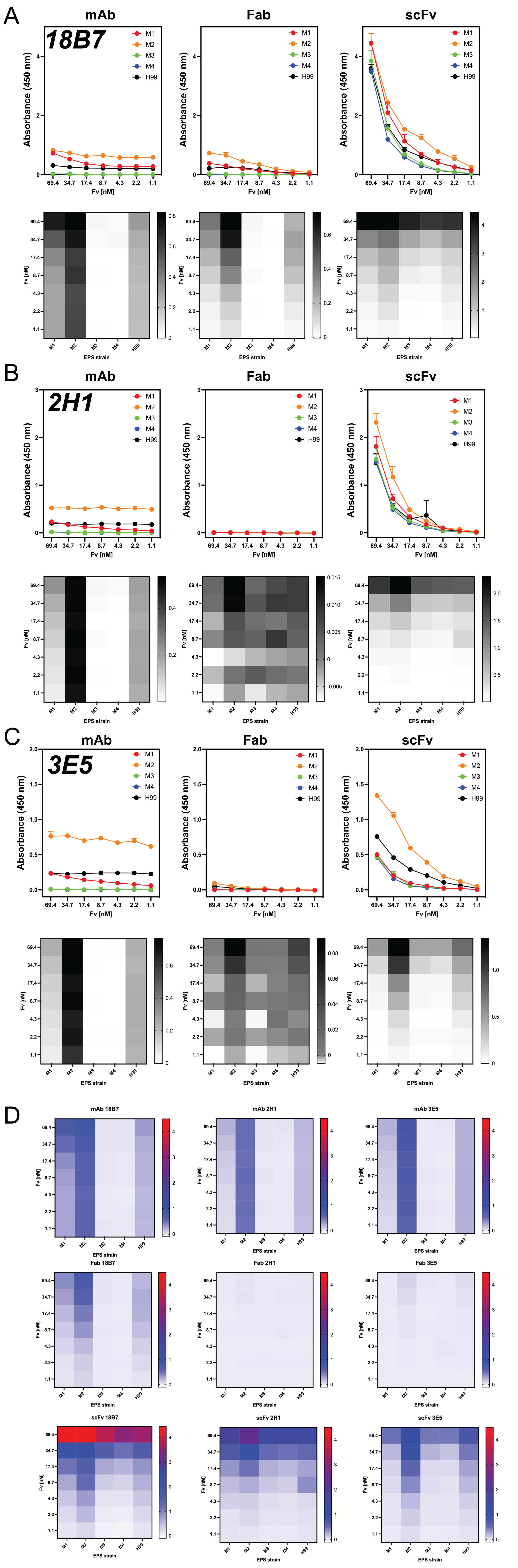


# Supplementary Fig. 5. Full exopolysaccharide specificity data (associated with Figure 4).

Full indirect ELISA quantification of binding across the full concentration gradient of antibody or antibody fragment. The concentration of antibody was at the highest concentration was 5 µg/mL and was normalized on a molar basis relative to Fv for antibody fragments, such that [mAb] = 5 µg/mL = 34 nM, [Fv] = 69 nM, [Fab]= 3.32 µg/mL = 69 nM, [Fv] = 69 nM, and [scFv] = 1.97 µg/mL = 69 nM, [Fv] = 69 nM. Plates were coated with EPS antigen from each single-motif strain at 1 µg/mL. For each family, the optical density from each experiment was normalized to an internal control. ODs are plotted in the top panels as line graphs with the same scale for each Ab group, and scaled individually for each antibody fragment in the bottom panels as a heat map. Data corresponding to 18B7, 2H1 and 3E5 families are presented in panels A, B, and C, respectively. D) Summary heat maps of normalized binding values, plotted on the same global scale, are presented. Heat map scale ranges from high binding (red) to intermediate binding (blue) to low binding (white).

# Table S1: EPS reactivity profiles (normalized OD values at 69.4 nm Fv for each Ab fragment) from indirect ELISA measurements to exopolysaccharide from each cryptococcal strain (associated with Figures 4 and S5).

|  |  | **Motif 1** | **Motif 2** | **Motif 3** | **Motif 4** | **H99** |
| --- | --- | --- | --- | --- | --- | --- |
| **mAb** | **18B7** | 0.72 ± 0.00 | 0.82 ± 0.02 | 0.02 ± 0.00 | 0.01 ± 0.00 | 0.31 ± 0.00 |
|  | **2H1** | 0.23 ± 0.00 | 0.52 ± 0.02 | 0.01 ± 0.00 | 0.01 ± 0.00 | 0.20 ± 0.00 |
|  | **3E5** | 0.23 ± 0.00 | 0.76 ± 0.04 | 0.00 ± 0.00 | 0.00 ± 0.00 | 0.23 ± 0.00 |
| **Fab** | **18B7** | 0.38 ± 0.00 | 0.73 ± 0.01 | 0.02 ± 0.02 | 0.00 ± 0.00 | 0.21 ± 0.04 |
|  | **2H1** | 0.00 ± 0.00 | 0.01 ± 0.00 | 0.00 ± 0.00 | 0.00 ± 0.00 | 0.00 ± 0.00 |
|  | **3E5** | 0.00 ± 0.00 | 0.09 ± 0.00 | 0.00 ± 0.00 | 0.00 ± 0.00 | 0.04 ± 0.00 |
| **scFv** | **18B7** | 4.45 ± 0.03 | 4.43 ± 0.23 | 3.84 ± 0.25 | 3.48 ± 0.07 | 3.59 ± 0.09 |
|  | **2H1** | 1.81 ± 0.15 | 2.31 ± 0.13 | 1.54 ± 0.16 | 1.48 ± 0.11 | 1.46 ± 0.14 |
|  | **3E5** | 0.50 ± 0.00 | 1.34 ± 0.01 | 0.46 ± 0.02 | 0.45 ± 0.02 | 0.75 ± 0.00 |


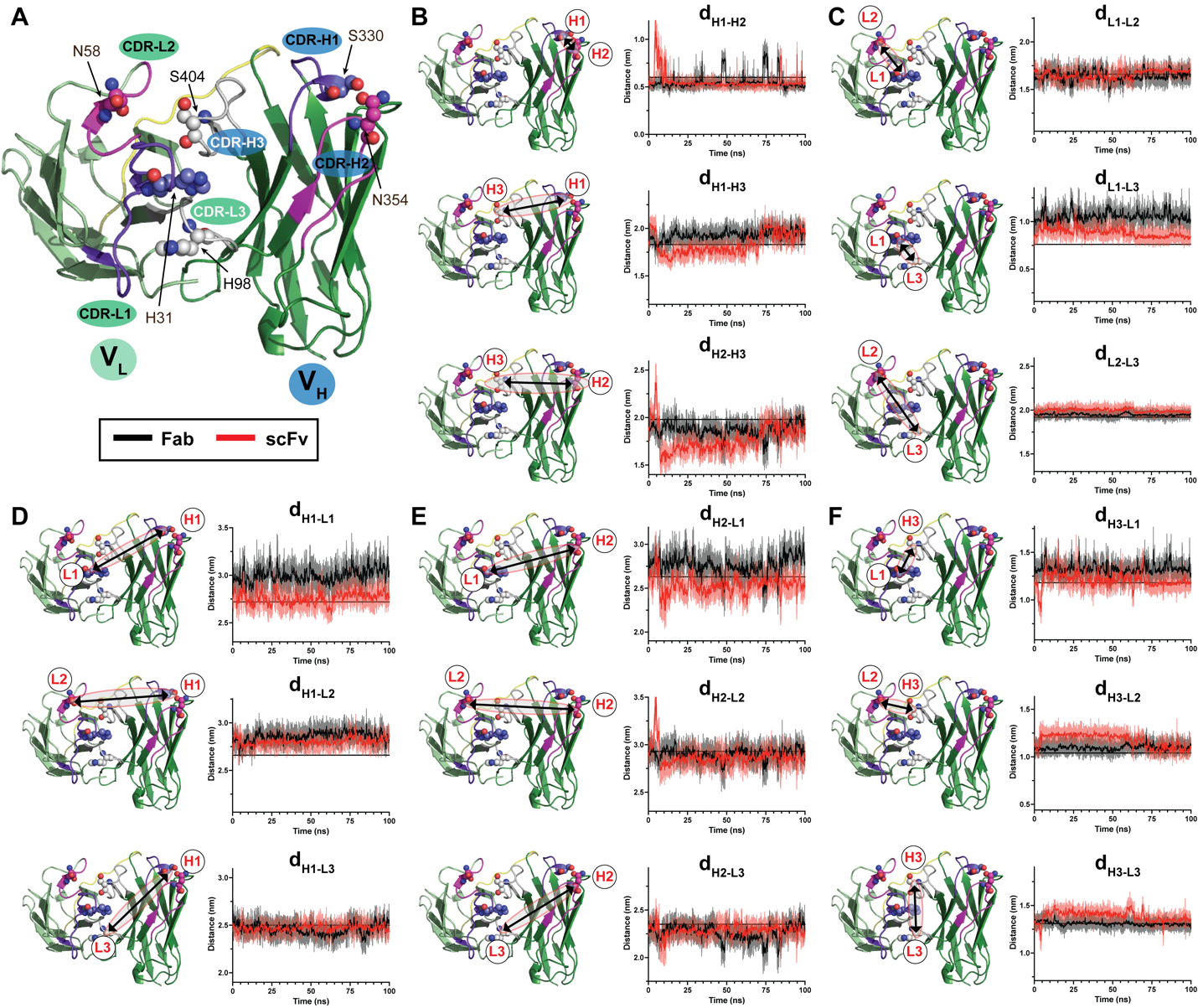


# Supplementary Fig. 6. Full distance measurements between all CDR pairs during MD simulations of mAb 2H1 Fab and scFv fragments.

Distances between CDRs (defined as distance between the Cα atom of a single central residue in each CDR) are measured across the MD trajectories. Distances measured from the original structure are overlaid as a black line. **A)** Structure of scFv 2H1 with CDRs highlighted and positions of residues used for distance measurements indicated. Intra-domain distance measurements over the simulation trajectory between **B)** heavy chain and **C)** light chain CDRs. Inter-domain distance measurements over the simulation trajectory between **D)** CDR-H1, **E)** CDR-H2, **F)** CDR-H3 and light chain CDRs. The raw data was smoothed with a zero-order polynomial, averaging 20 nearest neighbors, and superimposed onto the data to reveal general trends.


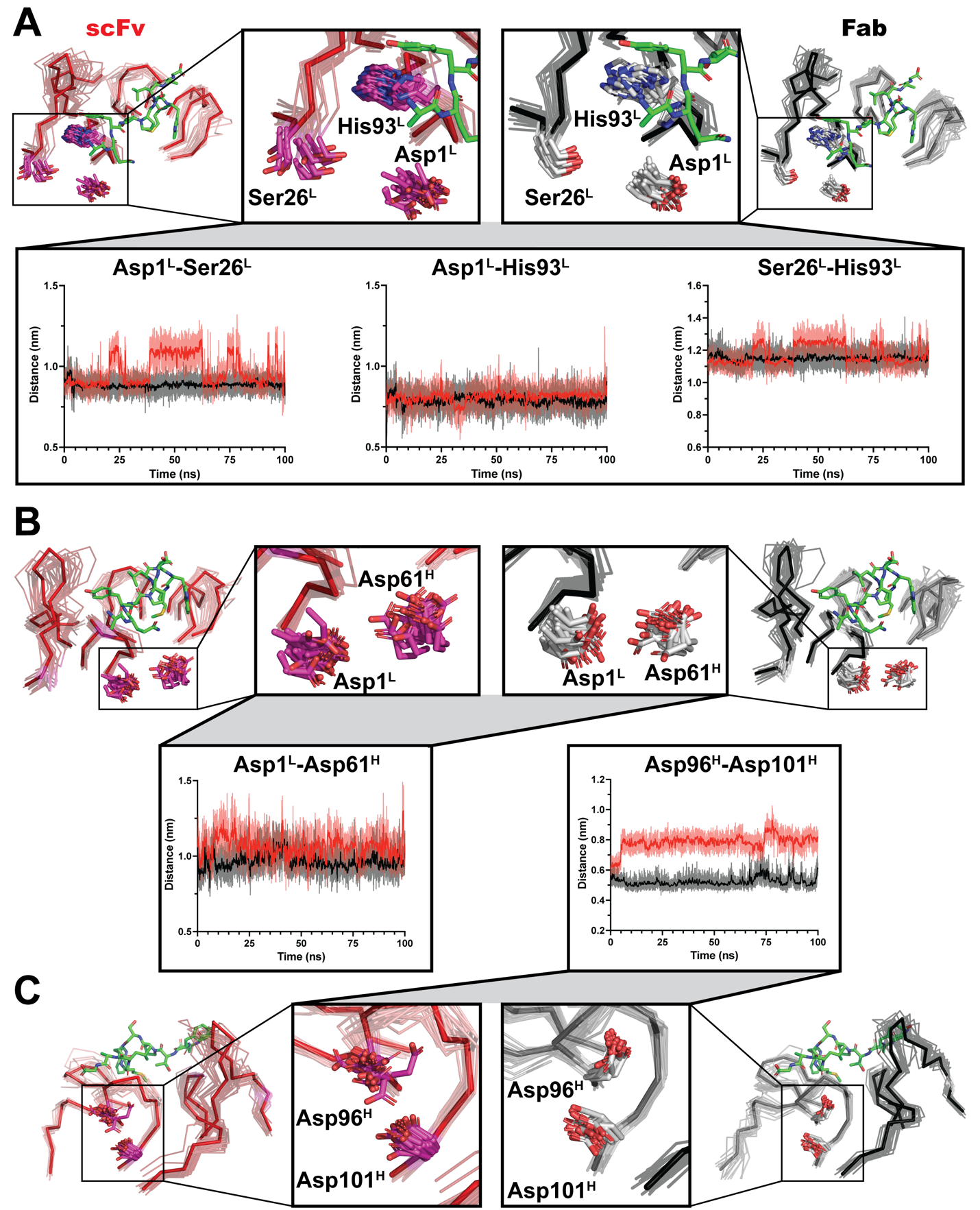


# Supplementary Fig. 7. Distance measurements between putative catalytic residues during MD simulations of mAb 2H1 Fab and scFv fragments.

Distances between putative catalytic residues in the paratope of mAb 2H1 (defined as distance between the Cα atoms) are measured across the MD trajectories. Backbone traces of the CDR loops for scFv (red) and Fab (grey) are depicted as ribbons and the conformations of each residue are depicted at magenta or gray sticks for scFv and Fab, respectively. For each pair or triad of residues, the location of the residues on the structural ensemble of Fab or scFv (colored red or black, respectively) is indicated with a black box and an expanded view of the configurations of the side chains is displayed. The distances for each pair over the simulation trajectory is shown for each pair, with data corresponding to scFv and Fab colored red and black, respectively. The raw data was smoothed with a zero-order polynomial, averaging 20 nearest neighbors, and superimposed onto the data to reveal general trends. **A)** Distances between the putative serine protease triad residues (D1-V_L_, S26-V_L_, H93-V_L_; Chothia numbering). **B)** Distance between aspartate residues of potential glycosidase site 1 (D1-V_L_, D61-V_H_ ; Chothia numbering). **C)** Distance between aspartate residues of potential glycosidase site 2 (D96-V_H_ , D101-V_H_; PBD numbering).

# Supplementary References:

1. Crawford, C.J., Guazzelli, L., McConnell, S.A., McCabe, O., d’Errico, C., Greengo, S.D., Wear, M.P., Jedlicka, A.E., Casadevall, A., and Oscarson, S. (2023). Synthetic Glycans Reveal Determinants of Antibody Functional Efficacy against a Fungal Pathogen. ACS Infect Dis. 10.1021/ACSINFECDIS.3C00447.

2. Young, A.C.M., Valadon, P., Casadevall, A., Scharff, M.D., and Sacchettini, J.C. (1997). The three-dimensional structures of a polysaccharide binding antibody to cryptococcus neoformans and its complex with a peptide from a phage display library: Implications for the identification of peptide mimotopes. J Mol Biol *274*, 622–634. 10.1006/jmbi.1997.1407.

3. Eryilmaz, E., Janda, A., Kim, J., Cordero, R.J.B., Cowburn, D., and Casadevall, A. (2013). Global structures of IgG isotypes expressing identical variable regions. Mol Immunol *56*, 588–598. 10.1016/J.MOLIMM.2013.06.006.
